# A Novel Rho-Like Protein TbRHP Is Involved in Spindle Formation and Mitosis in Trypanosomes

**DOI:** 10.1371/journal.pone.0026890

**Published:** 2011-11-11

**Authors:** Kanwal Abbasi, Kelly N. DuBois, Joel B. Dacks, Mark C. Field

**Affiliations:** 1 Department of Pathology, University of Cambridge, Cambridge, United Kingdom; 2 Department of Cell Biology, University of Alberta, Edmonton, Canada; University of Texas-Houston Medical School, United States of America

## Abstract

**Background:**

In animals and fungi Rho subfamily small GTPases are involved in signal transduction, cytoskeletal function and cellular proliferation. These organisms typically possess multiple Rho paralogues and numerous downstream effectors, consistent with the highly complex contributions of Rho proteins to cellular physiology. By contrast, trypanosomatids have a much simpler Rho-signaling system, and the Trypanosoma brucei genome contains only a single divergent Rho-related gene, TbRHP (Tb927.10.6240). Further, only a single RhoGAP-like protein (Tb09.160.4180) is annotated, contrasting with the >70 Rho GAP proteins from *Homo sapiens*. We wished to establish the function(s) of TbRHP and if Tb09.160.4180 is a potential GAP for this protein.

**Methods/Findings:**

TbRHP represents an evolutionarily restricted member of the Rho GTPase clade and is likely trypanosomatid restricted. TbRHP is expressed in both mammalian and insect dwelling stages of *T. brucei* and presents with a diffuse cytoplasmic location and is excluded from the nucleus. RNAi ablation of TbRHP results in major cell cycle defects and accumulation of multi-nucleated cells, coinciding with a loss of detectable mitotic spindles. Using yeast two hybrid analysis we find that TbRHP interacts with both Tb11.01.3180 (TbRACK), a homolog of Rho-kinase, and the sole trypanosome RhoGAP protein Tb09.160.4180, which is related to human OCRL.

**Conclusions:**

Despite minimization of the Rho pathway, TbRHP retains an important role in spindle formation, and hence mitosis, in trypanosomes. TbRHP is a partner for TbRACK and an OCRL-related trypanosome Rho-GAP.

## Introduction

The Rho subfamily of Ras-like GTPases are required for a multitude of cellular functions, including regulation of the actin cytoskeleton, participation in signaling pathways and cell cycle modulation [1]. The size and complexity of the Rho subfamily is rather variable between organisms; in *Homo sapiens* there are over twenty members, while *Saccharomyces cerevisiae* possesses only six members [2]. Also the number of Rho GTPases encoded by a given genome does not necessarily directly correlate with organismal tissue or cell-cycle complexity; for example, the metazoan *Caenorhabditis elegans* possesses only five Rho-class GTPases. The functional complexity associated with Rho proteins can be further extended by promiscuous interactions with GTPase-activating proteins (GAPs), guanine nucleotide exchange factors (GEFs), guanine-nucleotide dissociation inhibitors (GDIs), kinases and other factors. The RhoGAP family can be particularly complex and in *H. sapiens* at least 70 RhoGAP proteins are known, with complex domain architectures and expression profiles [3].

The complexity, status and importance of Rho-mediated signaling in organisms beyond the supergroup Opisthokonta, i.e. animals and fungi, is less well documented. In higher plant genomes, considerable Rho gene families are present; for example *Arabidopsis thaliana* has over ten Rho-like proteins, and many of these, the Rop proteins, arose in a lineage-specific manner [4]. Further, the amoebae *Dictystellium discoidium* and *Entamoeba histolytica* also possess substantial Rho-related families [5], [6]. Much of the variability of Rho repertoire between taxa is due to lineage-specific expansions within specific Rho subfamilies, implying that the ancestral composition was probably rather limited [7]. Finally, the number of Rho proteins and associated GAPs and GEFs encoded by the genome has been estimated for several protists of the Chromalveolata and Excavata supergroups; here Rho functionality appears de-emphasized, with only two putative Rho-like proteins in trypanosomes and one in apicomplexa [8].

The African trypanosome, *Trypanosoma brucei*, is a comparatively well characterized representative of the Excavata, and highly divergent from animals and fungi [9]. Characterization of *T. brucei* small GTPases are also advanced, but restricted almost exclusively to the members involved in intracellular transport, specifically Rabs and ARFs (reviewed in [10]). Rather less is known concerning functions of trypanosomatid Rho proteins. However, many aspects of the structure and functions of the cytoskeleton are well understood, and the predominance of the tubulin-based systems are well established [11]. In parallel with mammals and yeasts actin functions in clathrin-mediated endocytosis but in contrast there is no evidence for recognizable F-actin, and immunolocalization suggests predominance of short actin polymers or soluble G-actin [12]. Significantly, *T. brucei* possesses only two myosin genes, a myosin I orthologue and a novel trypanosomatid-specific myosin, a somewhat restricted repertoire consistent with the reduced role of actin. An identical configuration is present in *Leishmania major* but *Trypanosoma cruzi* possesses six lineage-specific myosins [13]. Despite this reduced actinomyosin system, all three kinetoplastida lineages possess considerable dynein and kinesin families of similar complexity to higher eukaryotes [14], [15].

In *T. cruzi* two Rho-related proteins are encoded in the genome. The first of these, TcRho1, has been partly characterized and, together with a function in life cycle progression and differentiation, evidence from heterologous expression suggests that TcRho1 can interact with the mammalian actin cytoskeleton in cell adhesion and migration assays; hence TcRho1 is likely a true Rho protein [16], [17], [18]). The role in metacyclogenesis may also reflect a function in cytoskeletal and morphological remodeling, consistent with classical Rho functionality. However, an orthologue for TcRho1 is absent from *T. brucei* and the gene is absent from the syntenic locus in both *T. brucei* and *L. major*. An additional gene encoding a divergent Rho-like protein, TbRHP, has been reported in *T. brucei*, and is shared among African and American trypanosomes and *Leishmania* [19]. The predicted TbRHP protein is somewhat larger than TcRho1, and preliminary *in silico* analysis indicated that TbRHP and TcRHP are highly divergent, *albeit* remaining *bona fide* members of the Rho subfamily [19]. The differences in the actinomyosin systems among *T. brucei*, *Leishmania* and *T. cruzi* may explain the need for two Rho-related proteins in the latter and would imply that RHP proteins perform a conserved function in these lineages.

As TbRHP is the sole member of the Rho family in African trypanosomes, we have sought to address the function of this protein by immunolocalization, RNAi-mediated knockdown, investigation of interaction partners, and detailed comparative genomics and phylogenetics. Evidence suggests a taxonomic distribution for TbRHP restricted to the trypanosomatids. We find that TbRHP is essential for mitosis, spindle formation and cytokinesis. Further, TbRHP interacts with TRACK, a previously identified mediator of cytokinesis in trypanosomes, and TbOCRL, the single RhoGAP protein encoded by trypanosomes [20].

## Methods

### Informatics

Identification of TbRHP and TcRho1 open reading frames (ORFs) have been described previously [18], [19]. Analysis of syntenic relationships between trypanosomatids was performed at tritrypdb.org. Rho, Ras and RLJ GTPase predicted protein sequences were obtained from a sampling of genomes from across the five available eukaryotic supergroups, with particular emphasis on the Excavata using BLAST [21], [22]. *Homo sapiens*, *Saccharomyces cerevisiae*, *Entamoeba histolytica*, *Phytophthora sojae*, *Arabidopsis thaliana*, *Dictyostelium discoideum*, *Naegleria gruberi*, *Trypanosoma cruzi*, *Trichomonas vaginalis*, *Trypanosoma brucei*, *Chlamydomonas reinhardtii* and *Giardia intestinalis* were sampled for the presence of the various homologues as described previously (Table S1) [23]. However, since genes from *Giardia* are rapidly evolving, potentially contribute to artifact in phylogenetic reconstruction and were not directly relevant to classification of the *T. brucei* RHP sequence, they were excluded. Sequences were aligned using ClustalX and manually adjusted [24]. Only regions of unambiguous homology were retained for phylogenetic analysis. This resulted in a dataset of 38 sequences and 139 amino acid positions (see supplementary material). Prot-test 1.3 determined that a Whelan and Goldman (WAG) amino acid transition matrix with a gamma correction for invariable positions and rate among sites best described the model of sequence evolution for the dataset. The optimal tree topology and posterior probability values were determined using MrBayes version 3.1.2 with 10^6^ MCMC generations, and burnin determined by graphical estimation and removal of all trees prior to the plateau [25]. Maximum likelihood bootstrap values were obtained on 100 pseudo-replicate datasets using PHyML implementing the parameters determined by Prot-test and using RAxML implementing a ProtCATWAG model. A similar procedure was used for analysis of the OCRL proteins, but in this instance protein domain searches were performed at CDDB.

### Cell lines and propagation

*T. brucei brucei* bloodstream form (BSF) and procyclic form (PCF) Lister 427 laboratory-adapted strains were cultured in HMI9 and SDM79 media respectively supplemented with 10% fetal bovine serum as described previously [26]. For RNA interference experiments, the tetracycline-responsive SMB cell line was cultured as described [26], [27]. For expression of epitope-tagged forms of TbRHP for localization, a single HA-tag was introduced between codons two and three at the N-terminus of the protein by PCR-mediated mutagenesis. The resulting HA-TbRHP was subcloned into pXS5, transfected and selected as described previously [28]. Expression of the transgene was validated by Western blotting.

### RNA interference

A 486 bp fragment of Tb10.70.0590 was selected and verified by RNAit software to specifically target the gene product in RNAi experiments [29]. This fragment was PCR-amplified from *T. brucei* genomic DNA using the following oligonucleotides AGCATCTGTAGTTGGGTGGG and CCCAATGCTCTTATGGGAGA, or GCTACTCGAGCTTGGATGTGAACGTGTTGG and CGTAGGATCCGGCATCAACGGTATTTCTTC and the product inserted into the p2T7TABlue plasmid or the p2T7 plasmid [30]. An AMAXA nucleofector was used to transfect tetracycline-responsive log-phase SMB cells with *NotI* digested p2T7•TbRHP following the manufacturer's procedure and as previously described [23]. For growth curves, cultures were inoculated to a concentration of 10^4^ cells ml^−1^ in triplicate with or without tetracycline at 1 µg ml^−1^. To monitor cell numbers cell density was assessed using a Z2 Coulter Counter (Beckman).

### Antibodies

Affinity purified antibody against TbRHP was raised in rabbits following expression of a full-length GST-TbRHP fusion protein in *Escherichia coli*. The full length protein was cloned downstream and in frame with GST into the BamHI site of pGEX6P. GST-TbRHP was expressed in BL21(DE3) transformed *E. coli* with pGEX6P•TbRHP in L-broth and induced with 1.0 mM isopropyl-β-D-thiogalactopyranoside. The majority of the fusion protein was insoluble, and hence for immunization SDS-PAGE-purified material (∼5 mg total) was used. Purity of the isolated GST-TbRHP band was estimated at >90% by SDS-PAGE and Coomassie Blue staining. Rabbits were immunized four times with a total of 5 mg recombinant protein in Freund's complete adjuvant (Covalab). For antibody affinity purification, GST-TbRHP was immobilized on cyanogen bromide-activated agarose (Sigma) following the manufacturer's procedure and antibody purified by standard methods; soluble GST-TbRHP corresponded to ∼20% of *E. coli* expressed protein. Purified antibody was stored in 50% glycerol/PBS at −20°C. Purified antibody was validated by Western blotting and competition assays using excess soluble GST-TbRHP. RNAi also confirmed the antibody to be specific for TbRHP (Figure S1 and data not shown). Antibody to trypanosome BiP was a gift from James Bangs and used as previously described [23], antibody to human histone H3, which cross-reacts with trypanosome H3 was from Abcam and used according to the manufacturers instructions. KMX1 antibody was a gift from Keith Gull and used as described [31].

### Protein electrophoresis and Western blotting

For Western analysis total lysates of 10^6^–10^7^ trypanosome cells were separated by 12% SDS-PAGE and transferred onto ImmobilonP (Millipore). Membranes were blocked and processed following standard procedures. The rabbit polyclonal TbRHP antibodies were used at a dilution of 1∶2000. All the other antibodies were at 1∶10000. Detection used enhanced chemiluminesence and exposure to X-ray film. Films were scanned and exposures quantitated using ImageJ software (NIH). Quantified data are expressed in arbitrary units and normalized to TbBiP, following reprobing, as a loading control.

### Immunofluorescence

Cells were grown to log phase, fixed in 3% paraformaldehyde (PFA) in Voorheis's modified PBS (vPBS) and adhered to poly-L-lysine slides (Sigma). For immunostaining, cells were permeabilized with 0.1% Triton X-100 and blocked in fetal bovine serum. Slides were incubated with antibodies as described previously [32] and mounted with Vectashield containing 0.4 µg ml^−1^ DAPI (4′,6-diamino-2-phenylindole dihydrochloride) to stain DNA (Vectalabs). Primary antibody working dilutions were 1∶500 for rabbit anti-TbRHP, 1∶1000 for KMX1, 1∶2000 for anti-BiP, 1∶500 for anti-GRASP, 1∶500 for anti-p67 (gift from J. Bangs), neat for anti-BBA4 (gift from K. Gull), 1∶3000 for anti-GFP (gift from M.P. Rout), 1∶5 for anti-FAZ L3B2 (gift from K. Gull), 1∶200 for anti-C-Myc (GeneTex, inc.) and 1∶1000 for all other antibodies. For examining mitochondria, cells were incubated with Mitotracker CMX-Red (Molecular Probes) at 250 nM in normal media for 20 minutes. Cells were washed with vPBS and fixed as above. Cells were examined on Nikon Eclipse 400 epifluorescence microscope fitted with Hamamatsu CCD digital camera and optically matched filter blocks. Image acquisition was with Metamorph software (Molecular Devices, Version 6) and processing in Photoshop (Adobe Systems, Inc.). All quantitation was done using identical exposures as appropriate and using the raw data within Metamorph.

### Conconavalin A uptake

BSF cells were harvested at 800 *g*, washed in serum-free media, and resuspended at a final concentration of 5×10^5^ cells ml^−1^ in serum-free media supplemented with 1% BSA. Parasites were equilibrated at 37°C for 15 minutes before addition of fluorescein-conjugated Concanavalin A (ConA, Molecular Probes) to a final concentration of 5 µg ml^−1^. After 30 minutes, uptake of fluorophore was quenched by the addition of ice-cold PBS. Samples were washed thoroughly at 4°C to remove excess probe. ConA samples were prepared for immunofluorescence as described previously [27].

### Cell cycle analysis

Analysis of the distribution of the cell population across the cell cycle was performed by staining with DAPI as described [27], and analysing at least 200 cells for each time point considered.

### Electron microscopy

For transmission electron microscopy, cells were fixed in suspension by adding chilled 5% glutaraldehyde (TAAB) and 8% paraformaldehyde (Sigma) in PBS in a 1∶1 ratio to the growth medium containing trypanosomes. Cells were fixed on ice for 10 minutes, centrifuged at 10 000 rpm for 5 minutes in 2 ml microcentrifuge tubes, the supernatant carefully replaced with fresh fixative for a further 50 minutes without disturbing the pellet, rinsed in 0.1 M sodium cacodylate and post fixed in 1% osmium tetroxide (TAAB) in same buffer at room temperature for 1 hour. After rinsing in buffer cells were then dehydrated in an ethanol series, adding 1% uranyl acetate at the 30% stage, followed by propylene oxide and then embedded in Epon/Araldite 502 (TAAB) and finally polymerized at 60°C for 48 hr. Sections were cut on a Leica Ultracut T ultramicrotome at 70 nm using a diamond knife, contrasted with uranyl acetate and lead citrate and examined on a Philips CM100 transmission electron microscope.

### *In situ* tagging

Proteins of interest were tagged using a PCR-based C-terminal *in situ* tagging strategy using either the pMOTag4G, pMOTag4H or pMOTag43M vectors [33]. The full pMOTag4G template cassette contains *Aequorea victoria* green fluorescent protein (GFP) followed by a *trans*-splicing signal region (the intergenic region of α and β-tubulin) and an antibiotic resistance marker cassette. pMOTag4H contains a 3xHA epitope in place of GFP and pMOTag43M contains a 3xMyc epitope in place of GFP. The cassette was amplified by PCR using primers specific to the target ORF. The following primers were used: Tb927.3.3180TagF, TGGGAATGCTTCAGCAAGTGGTGAAAAGAACAATGCTCCACGGAATCCCTTCTCATTTGGTGCCTCTTCTGGGAATGCTGGTACCGGGCCCCCCCTCGAG, Tb927.3.3180TagR, ACTAAAGAAGGGTAGAAAACAAAGAAAACACCAAATAAGGTACCTGACGCAGCGGCAACACCACGTCGACTTGCTGGCGGCCGCTCTAGAACTAGTGGAT, TbAUK1TagF, TTATCTCCCAAACAATTTACAACCTCCCACTGGAAAGCGTCCGCGTCTCGATGCAGAGCCAACTGCAGGGAAAGAGAATGGTACCGGGCCCCCCCTCGAG, TbAUK1TagR, ACAATACAACTCATGCGGGGTAATGCCTAAAAGTGTTTTTCTCCCTTCCTTCACTTTTGCTTCTGTTGTGATTTTGGCGGCCGCTCTAGAACTAGTGGAT, Tb09.160.4180TagF, GCAGCAACTGCAGCAAGAGAGGGAGGATGCTTTGCGCTTCGTCGAGTGTTTTCTTGTTCCACCCCCAGCCGTGATATTGGGTACCGGGCCCCCCCTCGAG, Tb09.160.4180TagR, ACTTCAAGTACCACGCAATTATAAACATTGATAATTTTTTTTTTGAAAAAAGAAAAAGCAAATACACAACCCCTTGGCGGCCGCTCTAGAACTAGTGGAT, Tb09.211.0620(actin)TagF, AACAACCTTCCAGTCGATGTGGATAACGAAGAGTGAATACGACGAGTCGGGACCCAGCATCGTACACAGCAAATGCTTTGGTACCGGGCCCCCCCTCGAG, Tb09.211.0620TagR, ACAAAGATAATCAGAATAACAAATAAGCAAAAAGTAGGTAACCAAAGTGTCCTATGGTATACTAAATTTTTTTTTGGCGGCCGCTGTAGAACTAGTGGAT. Following transfection, 2.5 µg ml^−1^ of hygromycin was added to the cell culture and clones were screened by limiting dilution. Positive colonies were assayed for correct insertion and expression using PCR and/or Western blotting.

### Yeast two-hybrid screen of a *T. brucei* genomic library against TbRHP

As bait the full length coding region of TbRHP was amplified from Lister 427 *T. brucei* genomic DNA and cloned into the pGBKT7 plasmid of the Matchmaker system (Clontech). The pGBKT7-TbRHP construct was used to transform AH109 *Saccharomyces cerevisiae* and transcriptional activity of the bait was tested by growth in SD-Trp/-Leu/-His media supplemented with a range of concentrations of 3-amino-1,2,3-triazole (3-AT). A *T. brucei* genomic library (kind gift of Ralph Schwarz) was screened by transformation of AH109 yeast expressing BD-TbRHP, transformants were plated in SD -Trp/-Leu/-His media in the presence of 3-AT. After incubation for a period of 72–96 hours at 30°C, colonies were recovered and DNA from each colony was extracted and sequenced. In order to eliminate false positives, isolated library prey plasmids were transformed into Y187 yeast and crossed with AH109 yeast carrying either the empty plasmid or the bait plasmid; activation of the reporter gene was assessed by growth in SD -Trp/-Leu/-His, plus 3-AT.

### Quantitative real time (qRT)-PCR

RNA was extracted using the Qiagen RNeasy kit following the manufacturer's instructions. 1×10^8^ BSF (SMB) or 5×10^7^ PCF cells were used per extraction. cDNA synthesis was performed as previously described [34]. For qRT-PCR, 5 µl of cDNA was used in a 25 µl reaction including IQ SYBR Green Supermix (BioRad) with 0.4 µM gene-specific forward and reverse primers. qRT-PCR reactions were performed in a BioRad MiniOpticon real time PCR detection system. For the RhoGAP gene product the following primers were used: TCGCCTTCGTTGGTAATCTT (RG-RTR7) and TATGAGCAGCGACCACAAAC (RG-RTF8).

### Bacterial two hybrid analysis

Analysis of protein-protein interactions was tested using the system as described by [35]. In brief, full length coding sequences of interest were amplified from trypanosome genomic DNA and cloned in frame into either pKT25 or pUT18 and sequence verified. Control constructs consisted of empty vectors or pKT25-ZIP and pUT18-ZIP, which contain the leucine zipper domain of *S. cerevisiae* GCN4 to promote association of the expressed products of the plasmids. Plasmids were introduced into *E. coli* BTH101, selected and plated onto either LB-X-gal or MacConkey medium agar plates. Colonies were allowed to grow at 37°C for up to 72 hours to visualize the colorimetric reaction. For quantitative analysis permeabilized cells from overnight cultures were assayed with *o*-nitrophenol-β-galactoside as substrate and normalized to total protein in the extract as measured by Bradford assay.

## Results

### RHP is a novel Rho-like GTPase restricted to Kinetoplastida

Tb927.10.6240 (NCBI accession XP_822866) (TbRHP) was previously identified as an ORF encoding a predicted 44 kDa Rho-like GTPase [19]. TbRHP is moderately upregulated at the mRNA level in bloodstream form (BSF) stages [34]. Alignment of the predicted TbRHP protein sequence against representative mammalian Rho and Ras proteins indicates that TbRHP is clearly distinct, despite conservation of important sequence features (Figure S1). Partial conservation within switch I is found but the canonical GTP-binding motif, LWDTAGQE, is represented by LCDSSGSE, differing at four of eight residues. Most significantly the canonical, and functionally important, Rho insert present in most Rho family members is absent [36]. Several additional indels were also found and one is shared with *H. sapiens* H-Ras. The C-terminal hypervariable region is longer than typical for Rho, but does retain a CVIM prenylation motif (data not shown). Overall these observations confirm TbRHP as a divergent Rho-related GTPase.

**Figure 1:**
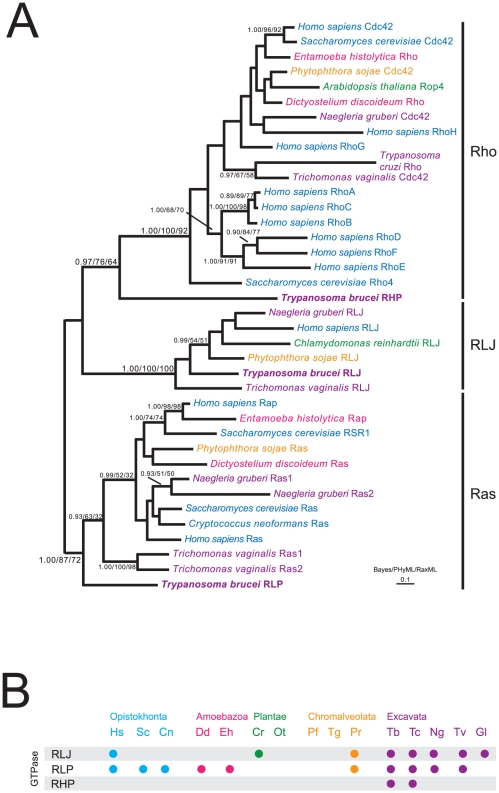
A novel Rho-related protein in trypanosomatids. (A) Bayesian reconstruction of Rho protein phylogeny. See methods for details. Numbers at internodes indicate statistical robustness and are for Baysian/PhyML/RaxML posterior probability, bootstrap and bootstrap support respectively. Clades are indicated by vertical bars and taxa colored according to supergroup membership: blue; Opisthokonta, pink; Amoebozoa, green; Viridiplantae, Orange; Chromalveolata and purple; Excavata. *T. brucei* sequences are highlighted in bold. (B) Comparative genomics representation of divergent Ras/Rho-related proteins across multiple taxa as determined by reciprocal BLAST and phylogenetic analysis. Taxa are colored as in (B) and two letter abbreviations are as in (B) plus Sc; *S. cerevisiae*, Cn; *C. neoformans*, Dd; *D. discoideum*, Eh; *E. histolytica*, Cr; *C. reinhardtii*, Ot; *O. tauri*, Pf; *P. falciparum*, Tg; *T. gondii*, Pr; *P. ramorum*, Tc; *T. cruzi*, Ng; *N. gruberi*, Tv; *T. vaginalis* and Gl; *G. lamblia*. RLP, RLJ and RHP denote Ras-like protein, Ras-like protein with DNAj domain and Rho-like protein respectively. Gray bars are for clarity.

A set of Rho, Ras and RLJ sequences were assembled, including at least one representative from each eukaryotic supergroup with a completed genome. Phylogenetic analysis places TbRHP at the base of the Rho clade but confidently excluded from the Ras and RLJ clades (Figure 1). Using standard BLAST/reverse BLAST procedures (see Methods) additional TbRHP orthologues could be identified only in African trypanosomes, *T. cruzi* and *Leishmania* (Figure 1, Table S1 and not shown). This contrasts to broader taxon representation for a novel small GTPase RLJ [37] and the Ras proteins (Figure 1). Given absence from additional excavate genomes (i.e. *Naegleria gruberi*, *Trichomonas vaginalis* and *Giardia lamblia*), we conclude that TbRHP is a novel GTPase likely restricted to the Kinetoplastida. Interestingly the *Leishmania* RHP orthologs are substantially larger (∼90 kDa) than the African and American trypanosome representatives (∼45 kDa), but retain C-terminal prenylation motifs and synteny, while in *T. congolense* a gene duplication has generated both a functional copy as well as a truncated version (Table S1 and data not shown). We suggest that TbRHP likely arose from a divergent ancestral trypanosomatid-specific gene in a lineage-specific manner and which may have been subject to somewhat accelerated evolution, rather than being an ancestral Rho lost from the vast majority of taxa. These data also suggest divergence in functionality between the trypanosomes and *Leishmania*. Further, this confirms distinct Rho configurations for *T. cruzi*, *T. brucei* and *L. major*, where TcRho has clear conserved Rho-like functions [17]. Significantly, restriction of a true Rho to *T. cruzi* suggests secondary loss from African trypanosomes and *Leishmania*.

### TbRHP is cytoplasmic

**Figure 2:**
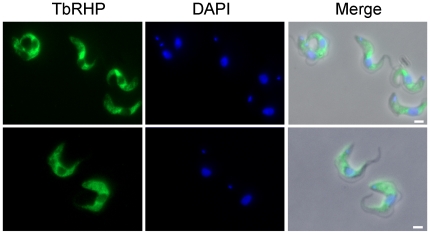
TbRHP is a cytosolic protein. BSF trypanosome cells expressing HA-TbRHP were prepared for immunofluorescence using a murine monoclonal anti-HA antibody followed by goat anti-mouse IgG FITC conjugate (green). DAPI was used to visualize DNA (blue). Left panels are HA fluorescence, middle panels DAPI and right merge with the phase contrast image. Bars, 2 µm.

Affinity-purified rabbit polyclonal antibodies raised against full-length TbRHP recognize a single band of ∼45 kDa in lysates from both BSF and procyclic forms (PCF) at approximately equivalent levels (Figure S2) but fail to generate a specific signal in immunofluorescence (data not shown). Therefore the full length TbRHP ORF was amplified from *T. brucei* genomic DNA and the insert cloned in frame C-terminal to an HA epitope in vector pXS5. BSF trypanosomes were transfected with pXS5•HA-TbRHP. Cells were screened by Western blotting and clones expressing an HA-tagged product of 43 kDa selected, consistent with the molecular weight of TbRHP plus an HA epitope (data not shown). When these cells were analyzed by immunofluoresence, the tagged protein was located in the cytoplasm but excluded from the nucleus (Figure 2). Staining was non-homogenous and a significant concentration was frequently observed in a perinuclear region as well as likely a soluble pool, typical for prenylated GTPases that cycle between membrane bound and soluble states (Figure 2).

### TbTHP is required for proliferation

**Figure 3:**
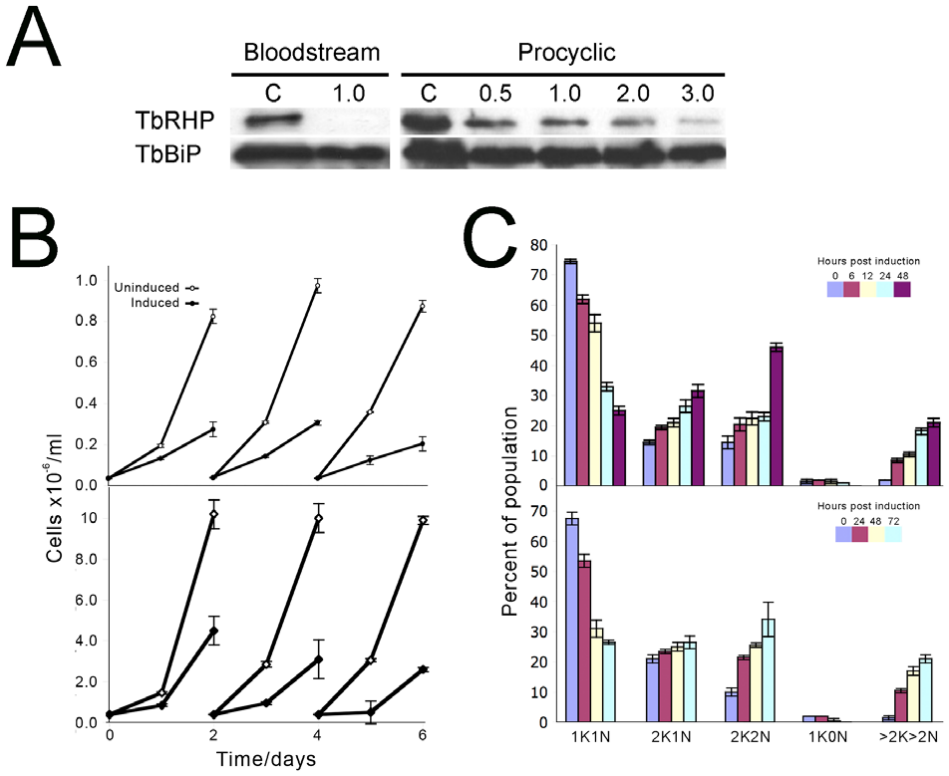
TbRHP is required for normal progression though the cell cycle. (A) Loss of TbRHP protein expression by RNAi. BSF and PCF cells were subjected to RNAi for TbRHP. Aliquots of culture were withdrawn at intervals and the abundance of TbRHP determined by Western blotting using affinity-purified rabbit polyclonal anti-TbRHP antibody followed by ECL detection. Equivalence of loading was determined by reprobing the membranes with antibody to trypanosome BiP (TbBiP). Numbers above the lanes indicate time, in days, while C indicates uninduced cells, equivalent to time zero. (B) Sawtooth growth curves for BSF (top) and PCF (bottom) cultures induced for RNAi against TbRHP. In both cases a growth defect is manifested within 24 hours, and is pronounced after two days. Filled symbols; induced cultures, open symbols; uninduced cultures. (C) Cell cycle progression is perturbed in both BSF and PCF cells by TbRHP RNAi. Logarithmic cultures of trypanosomes containing the relevant p2T7•TbRHP RNAi plasmid were induced and propagated as in (A). At the indicated times aliquots were withdrawn and the cells fixed, stained for DNA with DAPI and analyzed by fluorescence microscopy. Individual cells were scored for the number of nucleli (N) or kinetoplasts (K). At least 400 cells were analyzed at each time point and the analysis performed in duplicate. Error bars indicate the range and the values plotted are the mean.

We used RNA interference (RNAi) to suppress expression of TbRHP mRNA in BSF and PCF cells. TbRHP immunoreactivity in cell lysates was reduced in a time dependent manner following induction of dsRNA, validating both antisera and RNAi specificity (Figure 3). TbRHP became essentially undetectable in BSF cells after one day, while loss of TbRHP was rapidly evident in PCFs and reduced to ∼10% of normal levels after three days (Figure 3). Following induction proliferation was also decreased, suggesting an important role for TbRHP in cell cycle progression in both life stages (Figure 3). In both stages rapid decline in the proportion of normal interphase cells was found, i.e. possessing a single kinetoplast and nucleus (1K1N). This was accompanied by increased 2K2N cells and 2K1N cells, suggesting failure to complete cytokinesis. Furthermore, significant increases in the frequency of cells with aberrant numbers of nuclei and kinetoplasts was also observed (>2K>2N). The presence of these multi-nucleated cells suggests that S-phase and nuclear division proceed in a subpopulation of cells, but in the absence of complete cytokinesis. Early onset of proliferative defects, plus a rapid decrease in TbRHP levels, prompted us to examine turnover. When we treated BSF cells with cyclohexamide to block ongoing protein synthesis TbRHP was turned over with a half-life less than four hours, consistent with rapid emergence of the knockdown phenotype (Figure S2).

### TbRHP is required for correct nuclear segregation during mitosis

**Figure 4:**
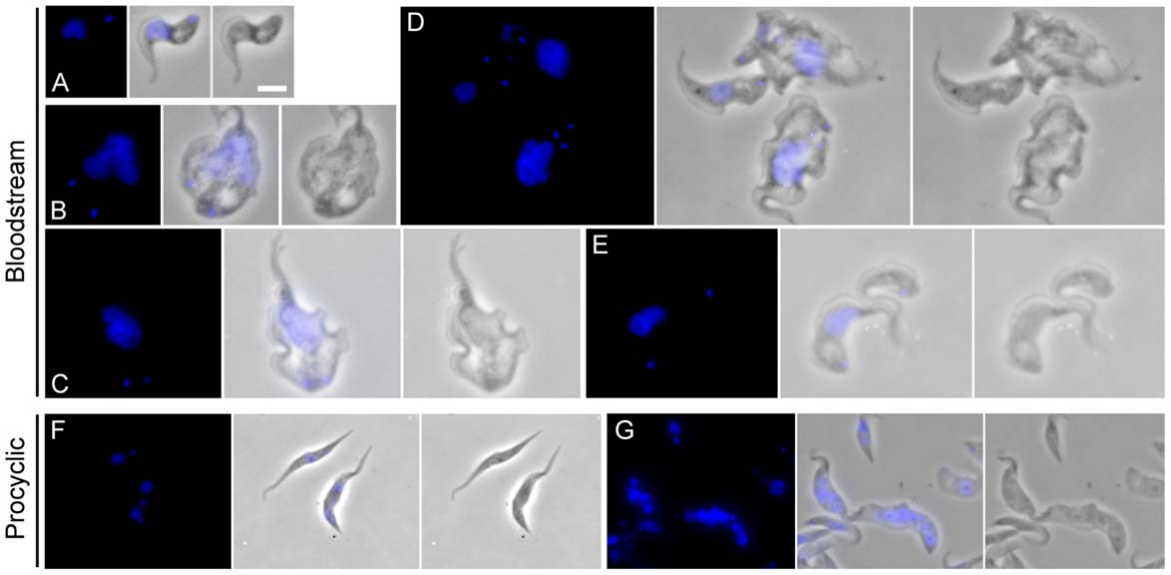
Gallery of morphotypes associated with RNAi of TbRHP in trypanosomes. (A and B) Uninduced BSF cells harboring the p2T7•TbRHP RNAi construct. (C–E) BSF cells induced for 24 hours, selected to illustrate the range of abnormal morphotypes, including multi-nucleate/multi-flagellar morphologies. The continued juxtaposition of apparently post-mitotic nuclei in these cells is evident in (C) and (D), suggesting a defect in migration following the completion of nuclear division. (F) Uninduced PCF cells harboring the p2T7•TbRHP RNAi construct. (G) PCF cells induced for 48 hours, selected to illustrate the fully segregated multinuclear phenotype. Each panel shows DAPI (blue) at left, phase contrast at right and channel merge at centre. Bar in (A), 3 µm.

In BSFs, in addition to the aberrant copy number of nuclei and kinetoplasts, major reorganisation of the overall cell morphology was evident (Figure 4, panels A–E), including cells with giardioid morphology possessing multiple flagella, an undulating membrane and a flattened appearance (Figure 4, panels C and D). This is likely a result of division of multiple cell bodies with incomplete cytokinesis. Moreover, many cells retained aberrantly sized nuclei, suggesting a failure of faithful mitosis but with ongoing DNA synthesis. However, kinetoplast replication and morphology were affected (Figures 3C and 4).

The morphology of PCF RNAi cells was distinct from the BSFs. While cells became distorted, this was less pronounced and manifest as increased diameter (Figure 4, panels F and G). Also, the presence of abnormally sized nuclei was not observed, but rather cells contained multiple nuclei with apparently normal volumes and shape. These data are consistent with the rather differing “checkpoint” control of PCF and BSF mitosis, but overall suggest that TbRHP is important for correct completion of mitosis, but not S-phase entry [38], [39].

### Distorted nuclei in TbRHP RNAi cells have normal compartmentalization and chromatin

**Figure 5:**
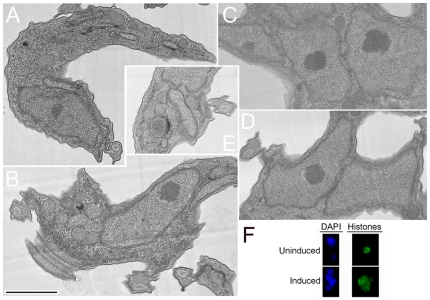
Nuclear architecture and chromatin appear normal in BSF cells depleted of TbRHP. (A–D) BSF parasites were induced for TbRHP RNAi for 24 hours and then fixed and processed for transmission electron microscopy. (A–D) Cells exhibited apparently normal nuclear architecture, including the double membrane nuclear envelope, nuclear matrix and nuceolus flagellar profile (A) and (B), normal spacing for the subpellicular microtubule array, and the absence of any obvious gross abnormalities. (E) some membranous structures are visible, which resemble accumulated membrane and possibly multi-vesicular bodies. These structures are rare and not seen in all cells. Bar, 1 µm. (F) Immunofluorescence analysis of histone and DNA distruibution. Anti-histone H3 antibody was used to highlight histones (green) and DAPI was used to visualize DNA (blue) in uninduced and induced cells. Representative images are shown.

We analyzed nuclei following TbRHP knockdown in more detail to determine if defective morphology and segregation are accompanied by a loss of normal chromatin structure. At the ultrastructural level we failed to observed significant defects in nuclear organization (Figure 5). Despite abnormal overall shape of TbRHP RNAi nuclei, the nucleolus, granular appearance of the nuclear matrix and the structure of the nuclear envelope all appeared normal (Figure 5, panel C).Therefore a major defect to nuclear organization is unlikely to explain abnormal segregation and the emergence of irregularly sized, juxtaposed nuclei in TbRHP RNAi cells. We did observe emergence of membrane-bound organelles that appeared to contain membranous inclusions in several cases (Figure 5, panel E); these structures are similar to multi-vesicular bodies [28] and may result from activation of autophagic or stress-related catabolic processes. Other organelles including the subpellicular microtubule array and flagellum appear normal. Therefore TbRHP is unlikely to be important in maintenance of basic organellar structure, but is required for correct nuclear segregation. We also stained cells with DAPI and a commercial anti-histone H3 antibody to visualize DNA and chromatin (Figure 5F). Despite the clear accumulation of multiple nuclei, histone distribution was only slightly impacted following RNAi against TbRHP, and again the nucleolus of each daughter nucleus was clearly observable, confirming that nuclear structure was largely unaffected.

### TbRHP is not required for correct division or segregation of non-nuclear organelles

**Figure 6:**
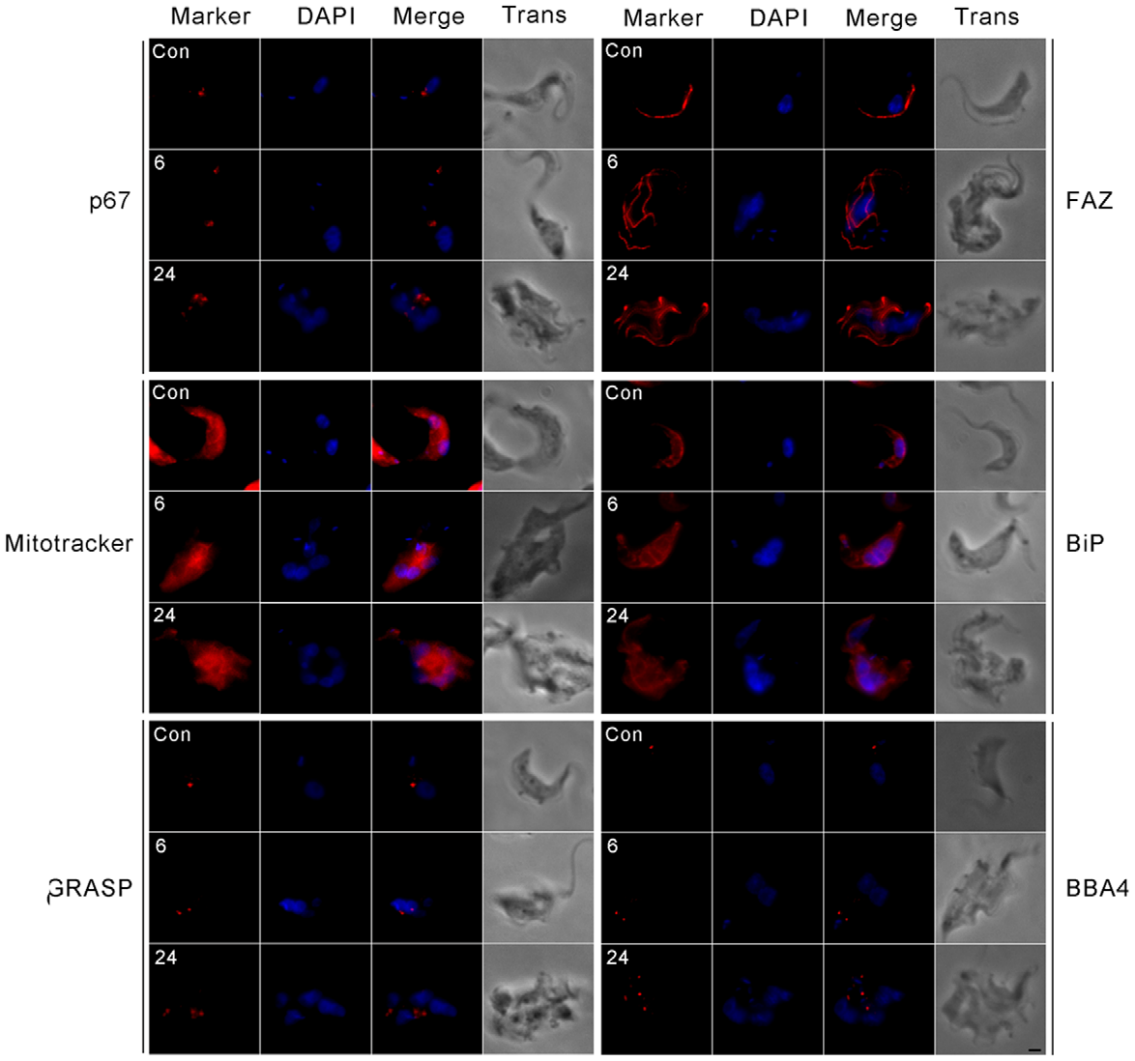
Cellular organelles have normal morphology and appear to replicate and divide normally following TbRHP RNAi. Lysosomes (p67), Golgi complex (GRASP), mitochondria (Mitotracker), endoplasmic reticulum (BiP), basal bodies (BBA4), and flagella (FAZ) were inspected in knockdown cells compared to control cells at 6 hours and 24 hours after the induction of RNAi. All organelles examined appeared morphologically normal and appeared to divide normally with respect to the number of total nuclei in a cell. Bar, 2 µm.

Trypanosomes display strict regulation in timing the division and positioning of cytoplasmic organelles during cell division [40]. We examined replication and partitioning of multiple cytoplasmic organelles, specifically lysosomes, the Golgi complex, mitochondria, endoplasmic reticulum, basal bodies, and the flagellum; more than thirty cells were examined for each datum. Despite increasingly abnormal overall cell morphology following knockdown, the morphology of organelles and their locations within the cell appeared normal (Figure 6). Additionally, organelles with distinct copy number, i.e. the Golgi complex, lysosome, basal bodies, and the flagellum maintained normal numbers with respect to the number of nuclei and kinetoplasts (Figure 6). For example, basal body and Golgi complex replication are closely associated with kinetoplast division and these organelles divide and migrate near coincidentally [41], [42]. The basal bodies and Golgi complexes were observed in similar number as the kinetoplast and in the expected position (Figure 6). These data suggest continued cytoplasmic organelle division and positioning in the absence of complete cytokinesis, ruling out a major and general defect in cytoskeletal functions.

### TbRHP expression is required for formation of a normal mitotic spindle

Failure of BSFs knocked down for TbRHP to correctly replicate and segregate the nucleus during cell division could be due to a nucleoskeletal defect, a known role of Rho GTPases, while the absence of major defects in organellar morphology or subpellicular microtubules plus faithful organellar division and segregation suggests a rather specific site of action (Figures 4, 5 and 6). In mammalian cells RhoA is implicated in the control of spindle formation *via* interactions with Aurora kinase (AUK), and similar roles for AUK and AUK-binding tousled-like kinase (TLK) have been described in *T. brucei* [31].

**Figure 7:**
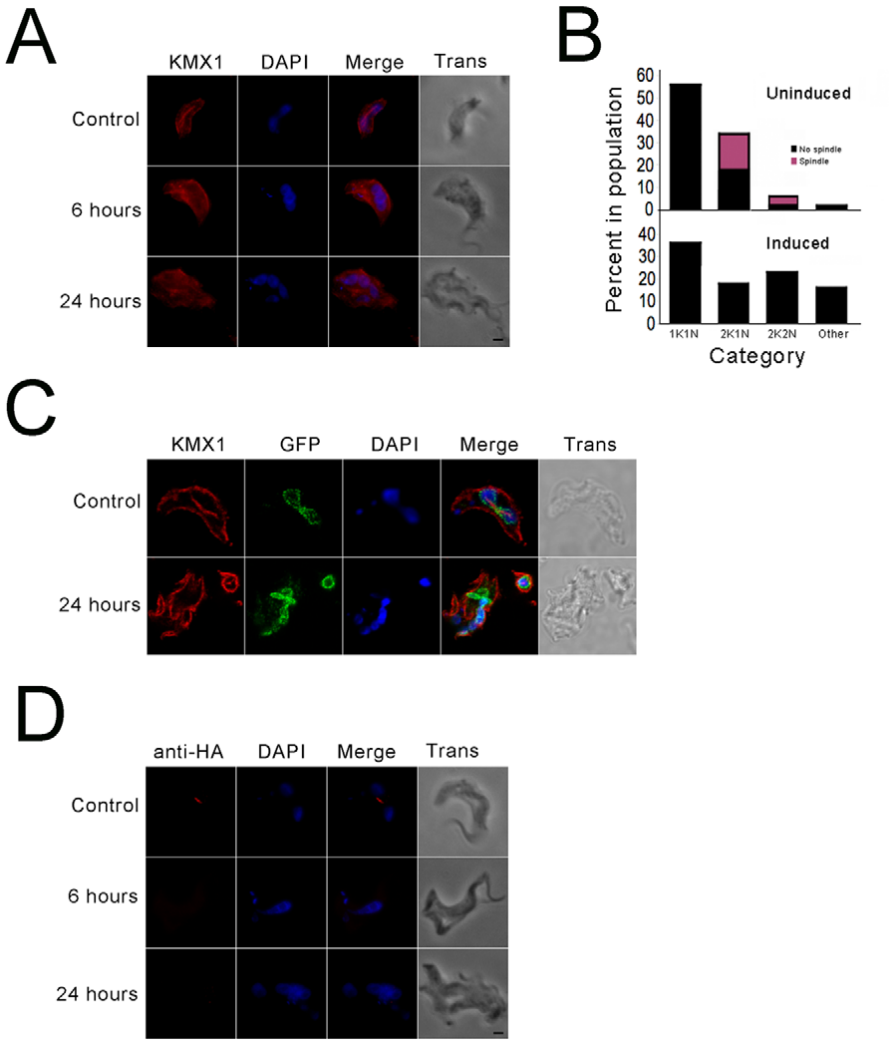
Suppression of TbRHP leads to spindle abnormalities. (A) BSF trypanosomes after 0 (control), 6, or 24 hours TbRHP RNAi induction were probed with anti-β-tubulin antibody (KMX1, red). DAPI was used to visualize DNA (blue). Note the presence of a clear spindle in the control cells and the complete absence of any large tubulin structure associated with the nucleus in the induced cells. Bar, 2 µm. (B) Quantitation of proportion of BSF cells with detectable spindles. Top panel shows uninduced cells, where the population is segregated by position in the cell cycle. Lower panel following 24 hours induction for TbRHP RNAi. K; kinetoplast, N; nucleus. n>200 for each analysis. (C) TbRHP RNAi was induced for 0 (control) or 24 hours in cells expressing TbNUP98-GFP (green) and cells were probed with anti-β-tubulin antibody (KMX1, red). DAPI was used to visualize DNA (blue). Spindles and segregated nuclei were observed in control cells, while in RNAi induced cells no spindles were observed and nuclei remained juxtaposed, but they did form individual nuclear envelopes as evidenced by TbNUP98-GFP fluorescence. (D) TbRHP RNAi was induced for 0 (control), 6, or 24 hours in cells expressing TbAUK1-HA. Cells were probed with an anti-HA antibody. TbAUK1-positive spindles were clearly observed in control cells but were completely absent in TbRHP depleted cells at 6 and 24 hours after induction. Bar, 2 µm.

To address the possibility that TbRHP elicits abnormal spindle behavior, cells were stained using KMX-1 monoclonal antibody against β-tubulin and counterstained with DAPI (Figure 7A). We observed a spindle in many cells in the control cell population, and significantly ∼50% of cells entering or in the mitotic phase contained a detectable spindle (Figure 7B). However, in cells depleted for TbRHP we never observed a spindle.

Further, we knocked down TbRHP in a BSF cell line with *in situ* tagged TbNUP98, a component of the nuclear pore complex, to highlight the nuclear periphery and also stained with KMX-1 antibody to visualize spindles. Using confocal microscopy we could clearly observe spindles and segregated nuclei in control cells, but in cells depleted of TbRHP, while daughter nuclei appeared to form individual nuclear envelopes, they often remained juxtaposed and did not segregate, while no spindles were observed (Figure 7C and data not shown). To validate this absence of detectable spindles TbRHP RNAi was induced in a BSF cell line with *in situ* tagged TbAUK. No spindles were seen in those cells displaying the RNAi-induced phenotype even when nuclei were apparently dividing (Figure 7D).

### TbRHP interacts with TbOCRL, a Rho-GAP

**Figure 8:**
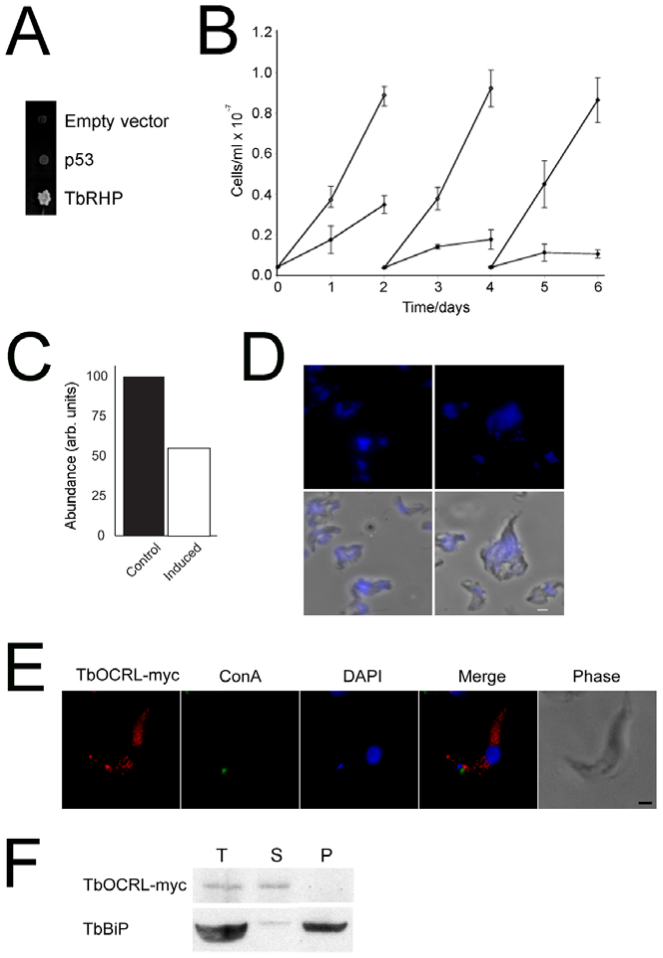
TbOCRL interacts with TbRHP. (A) Yeast two hybrid mating assay showing growth for TbRHP when mated with cells harboring TbOCRL plasmid. Growth is not obtained with either the empty vector or an irrelevant insert (p53). (B) Growth curve for cells induced for TbOCRL RNAi. Open symbols are uninduced cells and closed symbols the induced cells. Insert shows quantitation for TbOCRL mRNA levels by qRT-PCR at 24 hours post-induction. (C) Cells induced for TbOCRL RNAi, demonstrating the multinucleated phenotype and disruption of normal cell morphology. DAPI was used to visualize DNA (blue). (D) Localization of myc-tagged TbOCRL. TbOCRL-myc (red) was observed in a diffuse cytoplasmic location with distinct puncta. These puncta did not co-localize with the flagellar pocket as marked by Concanavalin A (ConA, green). DAPI was used to visualize DNA. Bar, 2 µm. (E) TbOCRL is soluble. Trypanosomes expressing the TbOCRL-myc tag fusion protein were subjected to hypotonic lysis in 50 mM Tris-HCl pH 7.4 plus protease inhibitors, soluble and insoluble fractions separted by centrifugation and equivalent aliquots separated by SDS-PAGE and transferred to PVDF membrane. The membrane was probed with anti-myc antibody and anti-TbBiP as loading control and pellet marker. T; total lysate, S; soluble fraction, P; pellet fraction. Essentially all TbOCRL-myc reactivity was recovered in the soluble fraction.

We searched for interaction partners of TbRHP both *in silico* and using a yeast two hybrid screen. The trypanosome genome possesses only one confidently predicted RhoGAP domain-containing open reading frame (ORF), Tb09.160.4180. A yeast two hybrid screen using TbRHP as bait confidently identified only two partners, the predicted RhoGAP and a second putative partner Tb11.01.3170/3180, TbRACK1. We confirmed the TbRHP:Tb09.160.4180 interaction on triple dropout plates (Figure 8A), suggesting *bona fide* interaction. Tb09.160.4180 is a member of the multi-domain occulo-renal-cerebro syndrome of Lowe (OCRL) family, and orthologues are present in other trypanosomatids, amoeba, metazoa and many fungi (Figures S3 and S4, [43]), but are absent from plants and chromalveolates [43]. Tb09.160.4180 carries a conserved RhoGAP domain R to Q mutation predicted to inactivate GAP activity (Figure S2, [44]). We designate Tb09.160.4180 as TbOCRL.

RNAi against TbOCRL (validated by qRT-PCR (Figure 8)) indicated profound effects on BSF proliferation and disorganized morphology, including multiple incompletely segregated nuclei and giant cells (Figure 8C and 8D). Broadly, TbOCRL RNAi phenocopies TbRHP RNAi, consistent with acting in the same pathways. We localized *in situ* tagged TbOCRL to a cytoplasmic location but excluded from the nucleus and similar to TbRHP (Figure 8E). Interestingly TbOCRL also localized to one or two puncta at the tip of the cell or near but not at the flagellar pocket pocket (Figure 8E). This is also similar to the localization of *H. sapiens* OCRL, which is present at the *trans*-Golgi network (TGN), early endosomes and as diffuse and punctate cytoplasmic pools [45]. Further, TbOCRL was found in the soluble pool following hypotonic lysis, suggesting that the protein has a presence in the cytosol and hence is likely to be able to interact with soluble TbRHP *in vivo* (Figure 8F).

### TbRACK1 interacts with TbRHP

**Figure 9:**
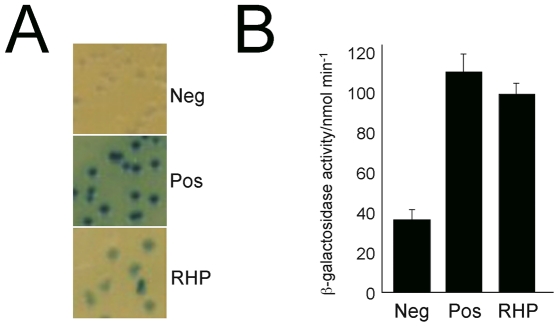
Validation of TbRHP and TbRACK interaction. (A) Bacterial two hybrid mating assay for interaction between TbRHP and TbRACK (Tb11.01.3180). Blue colonies are detected on beta-Gal plates after 48 hours growth in *E. coli* for both a positive control and TRACK. (B) Quantitation of galactosidase activity in cells harboring the TbRHP bait plasmid and a negative control (irrelevant insert) or TRACK (Tb11.01.3180) and a positive control pair of plasmids.

The second TbRHP interaction partner identified from the two hybrid screen, TbRACK1, is the *T. brucei* orthologue of receptor for activated C-kinase 1 (RACK1), a WD40 β-propeller protein that interacts with receptors, signaling molecules, and heterotrimeric GTPases. TbRACK1 knockdown led to defective morphology and cytokinesis defects, and the protein has recently been implicated as a ribosomal component and eIF1A-interacting factor [20], [46]. We used bacterial two hybrid to further validate the TbRHP-TbRACK1 interaction [35]. Evidence for interaction was seen only in the positive control and TbRHP and TbRACK1 pair (Figure 9A). A spectrophotometric galactosidase assay confirmed that TbRHP and TbRACK1 display significant interaction (Figure 9B). Taken together with identification by yeast two hybrid and known interactions between Rho and RACK in mammalian cells, this suggests an evolutionarily conserved interaction. Finally both TbRACK1 and TbRHP are both cytosolic, and therefore can also potentially interact *in vivo*.

## Discussion

Ras-like GTPases are ubiquitous mediators of signaling and control mechanisms essential for multiple aspects of cell function. Trypanosomatids have a restricted GTPase repertoire, but the full contributions of this superfamily to trypanosome cell biology and disease mechanisms remain undefined. Other than the Rab and ARF subfamilies, little is known. As *T. brucei* contains only a single Rho-related protein, TbRHP, we sought to understand the evolutionary history and possible cellular functions of this protein in some detail.

The TbRHP protein sequence is highly divergent and excluded from the Ras, RLJ and core Rho clade and restricted to trypanosomatids. *Leishmania spp*, other African trypanosomes and *T. cruzi* all possess TbRHP orthologues, while *T. cruzi* also possesses a conventional Rho, TcRho1 [16]. Absence of TcRho1 from the syntenic locus of *L. major* and *T. brucei* confirms TbRHP as the only Rho-like GTPase in these organisms. The presence of conventional Rho proteins in *T. vaginalis* and *T. cruzi* suggests that absence of a conventional Rho from African trypanosomes and *Leishmania* is due to secondary loss, while TbRHP probably arose uniquely in an ancestral trypanosomatid.

TbRHP is expressed in both major trypanosome proliferative life stages and required to complete cytokinesis. TbRHP RNAi results in rapid cessation of cell cycle progression, probably due to rapid turnover of the protein; this dynamic expression may facilitate a rapid response to TbRHP signaling. Significantly, the GTP-binding WDTAG motif is divergent in TbRHP, and may suggest altered GTPase activity. However, TbRHP does retain interaction with an OCRL family RhoGAP, suggesting that TbRHP can bind GTP as GAP proteins are specific for the GTP-bound form of small GTPases.

Progressive enlargement of cells and accumulation of incorrectly segregated nuclei is observed in the BSF form, while in the PCF stage multiple fully separated nuclei are observed. Therefore both life stages can apparently relicense for S-phase and that TbRHP is not required for this or S-phase completion, but the distinct morphologies that subsequently occur probably arise due to differential checkpoints in these stages and downstream events following S-phase and nuclear division. An obvious part of the mechanism here is the failure to build a normal spindle, as evidenced by both loss of KMX-1 staining and the absence of recruitment of AUK-1. However, previous work suggests that should the spindle completely fail, tetraploid cells with a single nucleus and a cytoplast (zoid) are generated [50]. This is clearly not the case here as neither cytoplasts nor cells with a single enlarged nucleus were observed, but rather multi-nucleated cells retaining equivalent numbers of nuclei and kinetoplasts. Replication, division and morphology of cytosolic organelles, including non-spindle tubulin structures such as the subpellicular array and flagellum, appear largely unaffected and continue even in the absence of complete cytokinesis.

The absence of cytoplasts indicates a potent block to cell division, i.e. absence of cytokinesis licensing, while the lack of severe alterations in nuclear architecture was also distinct from simple spindle ablation. While we cannot rule out a residual spindle, we can exclude a trivial explanation such as simple loss of KMX-1 immunoreactivity as AUK-1 also failed to be recruited. Hence any residual spindle or similar structure is likely significantly abnormal, but we cannot conclude complete ablation. Recently a tubulin-independent, actin-dependent nuclear scission process was reported in *Schizosaccharomyces pombe* which results in morphological nuclear defects and was suggested as a potential ancient/ancestral nuclear division mechanism [47]. PCF cells expressing GFP-actin possess a small actin bridge between dividing nuclei in mitotic and post-mitotic cells, which progressively thickens as the nuclei divide, suggesting that a similar mechanism could be operating here (Figure S5). However, further work is required to determine if residual tubulin structures or such a divergent mechanism are operating in the TbRHP knockdown cells.

**Figure 10:**
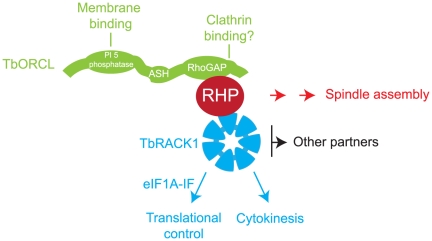
Model for TbRHP interactions and downstream functions. TbRHP and its two interacting partners are shown, TbOCRL as a series of green lozenges to indicate the multidomain structure of the protein and TbRACK as a seven-bladed structure reflecting the WD40 fold. Interactions between these factors are shown. Those interactions or functions that have been demonstrated in *T. brucei* are highlighted in red, while interactions inferred by homology with mammalian systems are shown in gray. The multiple interactions attributed to RACK1 in mammalian cells are shown as ‘Other partners’ for simplicity.

The functional roles of TbRHP were further extended by identification of TbOCRL and TbRACK1 as interaction partners. TbOCRL possesses a C-terminal RhoGAP domain and an N-terminal phosphatase domain, an architecture conserved with metazoan and amoebozoan OCRL [43], [44], [45], [48], [49], while TbRACK1 is a β-propeller protein (Figure 10). Both proteins interact with Rho in mammalian cells, indicating retention of at least this component of the Rho interactome across eukaryotes, and possibly representing an evolutionary conserved core [51]. No further Rho-GAP or GAP-related sequences were identified by two hybrid or trypanosome genome searches. TbOCRL retains an R to Q mutation in the RhoGAP domain and conserved phosphoinositide-binding residues; therefore TbOCRL is unlikely to act as a GAP but may target TbRHP to specific membrane microdomains and, by homology with the mammalian orthologue, to the clathrin-dependent endosomal system [52], [53], [54]. Importantly mammalian OCRL sits at the centre of an elaborate network of endocytic proteins [52], [54]. It is unclear if TbOCRL retains the clathrin-binding function of mammalian OCRL, and part of the downstream network is clearly divergent as APPL (adaptor protein containing PH and PTB domains and leucine-zipper motif) proteins, partners of mammalian OCRL and Rab5 effectors are absent from *T. brucei* [54]. Significantly, cytokinesis defects can occur when endocytic trafficking is disrupted, likely in part explaining the phenotypes of both TbOCRL and TbRHP knockdowns [55]. Significantly, no enlargement of the flagellar pocket was found, suggesting no major block in general endocytic trafficking [56]. TbRACK1, the second TbRHP partner, has been well characterized. Earlier work identified a role in cytokinesis, with TbRACK1 depleted cells exhibiting incomplete cytokinesis [20], somewhat similar to TbRHP. More recently TbRACK1 has been implicated in translational control, by association with polysomes [46]. Interestingly, TbRACK1 is not exclusively associated with the polysome fraction, and the highly complex interaction network of RACK in higher eukaryotes makes the complete repertoire of contributions of TbRHP-TbRACK1 interactions difficult to evaluate. However, overall these data do suggest that TbRHP sits at the centre of a network of interactions that touches on a large number of pathways.

In summary, TbRHP, the sole Rho-related GTPase in *T. brucei*, is essential for normal proliferation and cells lacking TbRHP fail to form a normal spindle or complete/license cytokinesis. TbRHP interacts with both TbOCRL/RhoGAP and TbRACK1, and knockdowns of both proteins produce similar phenotypes to TbRHP knockdown. Our data suggest a conserved Rho-like pathway in trypanosomes and unite TbRACK1, probable endocytic trafficking pathways and phosphoinositide signaling with a divergent trypanosomatid Rho-like GTPase. Therefore, despite high divergence and restriction to trypanosomatids, critical aspects of Rho functionality are retained by the TbRHP protein.

## Supporting Information

Figure S1 **Alignment of TbRHP with representative Rho protein sequences.** Predicted protein sequences were retrieved from the non-redundant database using BLASTp. Orthology was verified by reverse BLAST against the *T. brucei* genome sequence and selected sequences were aligned using ClustalX and default parameters. “-” represents gaps introduced into the alignment for optimization. Colorization indicates identity, conservative and semiconservative substitutions on a five point scale from red (conserved in 80% or more sequences), through light red, blue, light blue and white (no conservation).
(TIF)

Figure S2 **Characterization of anti-TbRHP antiserum and turnover of TbRHP.** (A) Western analysis of whole cell lysates prepared from BSF and PCF trypanosomes (B and P respectively); Whole cell lysates from 10^7^ cells were fractionated by SDS-PAGE and transferred to a nitrocellulose membrane. The blot was probed with affinity purified anti-TbRHP antibodies and signal was detected using ECL; a single band at ∼45 kDa was detected at equivalent intensity in both life stages. Numbers and bars at right indicate migration positions of co-electorphoresed molecular weight standards, in kDa. (B) Turnover of TbRHP in BSF cells. A log-phase BSF trypanosome culture was treated with cyclohexamide to inhibit new protein synthesis, and aliquots withdrawn from the culture at one hour intervals. Total lysates were fractionated by SDS-PAGE, transferred to nitrocellulose membrane and levels of TbRHP were monitored by Western blotting using affinity-purified anti-TbRHP antibodies. (C) Western blots were quantified using densitometry. TbRHP has a half-life of 3.5–4.0 hours. The data are a representative of three replicate experiments.
(TIF)

Figure S3 **Tb09.160.4180 is a representative member of the OCRL family.** Predicted protein sequences were retrieved from the non-redundant database using BLASTp. Orthology was verified by reverse BLAST against the *T. brucei* genome sequence and selected sequences were aligned using ClustalX and default parameters. “-” represents gaps introduced into the alignment for optimization, and “:”, “.” and “*” indicate semiconservative, conservative or identical amino acids respectively below the relevant system. Residues involved in inositol phosphate binding are indicated in bold and the position of the active arginine residue in the Rho GAP domain is shown bold underline, above the relevant system. Note that the *O. tauri* sequence is highly divergent within the Rho GAP domain, and is unlikely to be a member of the OCRL gene family.
(RTF)

Figure S4 **Phylogenetic reconstruction for OCRL family.** Taxa included are as in (A). Taxon abbreviations are: Ci; *Ciona intestinalis*, Dd; *Dictyostellium discoidium*, Hs; *Homo sapiens*, Lb; *Leishmania braziliensis*, Ot; *Ostreococcus tauri*, Rn; *Rattus norwegicus*, Tb; *Trypanosoma brucei*, Tc; *Trypanosoma cruzi* and Tr; *Tribolium castaneum*.
(TIFF)

Figure S5 **Actin localizes to the division plane between daughter nuclei in mitotic PCF cells.** In cells expressing actin-GFP, a thin bridge of actin was observed at the division plane between dividing nuclei (white arrows) in mitotic cells (2K1N and 2K2N). This band of actin appeared to progressively thicken as nuclei divided. Such a structure was not observed in non-mitotic (1K1N) cells.
(TIFF)

Table S1 **Accession numbers for sequences used in this study.** Top: Rho and Rho-related proteins included in reconstruction in Figure 1. Middle: OCRL proteins included in the analysis of OCRL phylogeny in Figure S2. Lower: TbRHP syntenic genes and orthologues from trypanosomes and *Leishmania* species.
(PDF)
