# Correction: A Novel Rho-Like Protein TbRHP Is Involved in Spindle Formation and Mitosis in Trypanosomes

**DOI:** 10.1371/annotation/b4b38a99-0b4c-483a-ad21-4dd721974b97

**Published:** 2011-11-29

**Authors:** Kanwal Abbasi, Kelly N. DuBois, Ka Fai Leung, Joel B. Dacks, Mark C. Field

The authors wish to add Ka Fai Leung as the third author to the manuscript. The updated byline is: Kanwal Abbasi1, Kelly N. DuBois1, Ka Fai Leung1, Joel B. Dacks2, Mark C. Field1*

The updated citation is: Abbasi K, DuBois KN, Leung KF, Dacks JB, Field MC (2011) A Novel Rho-Like Protein TbRHP Is Involved in Spindle Formation and Mitosis in Trypanosomes. PLoS ONE 6(11): e26890. doi:10.1371/journal.pone.0026890

Ka Fai Leung Performed the experiments, Contributed reagents/materials/analysis tools, and Wrote the Manuscript. 

