# Supplementary figures and images for: A Novel Rho-Like Protein TbRHP Is Involved in Spindle Formation and Mitosis in Trypanosomes
Source: PLoS One. 2011 Nov 11;6(11):e26890. doi: 10.1371/journal.pone.0026890 (PMC3214021; doi:10.1371/journal.pone.0026890)

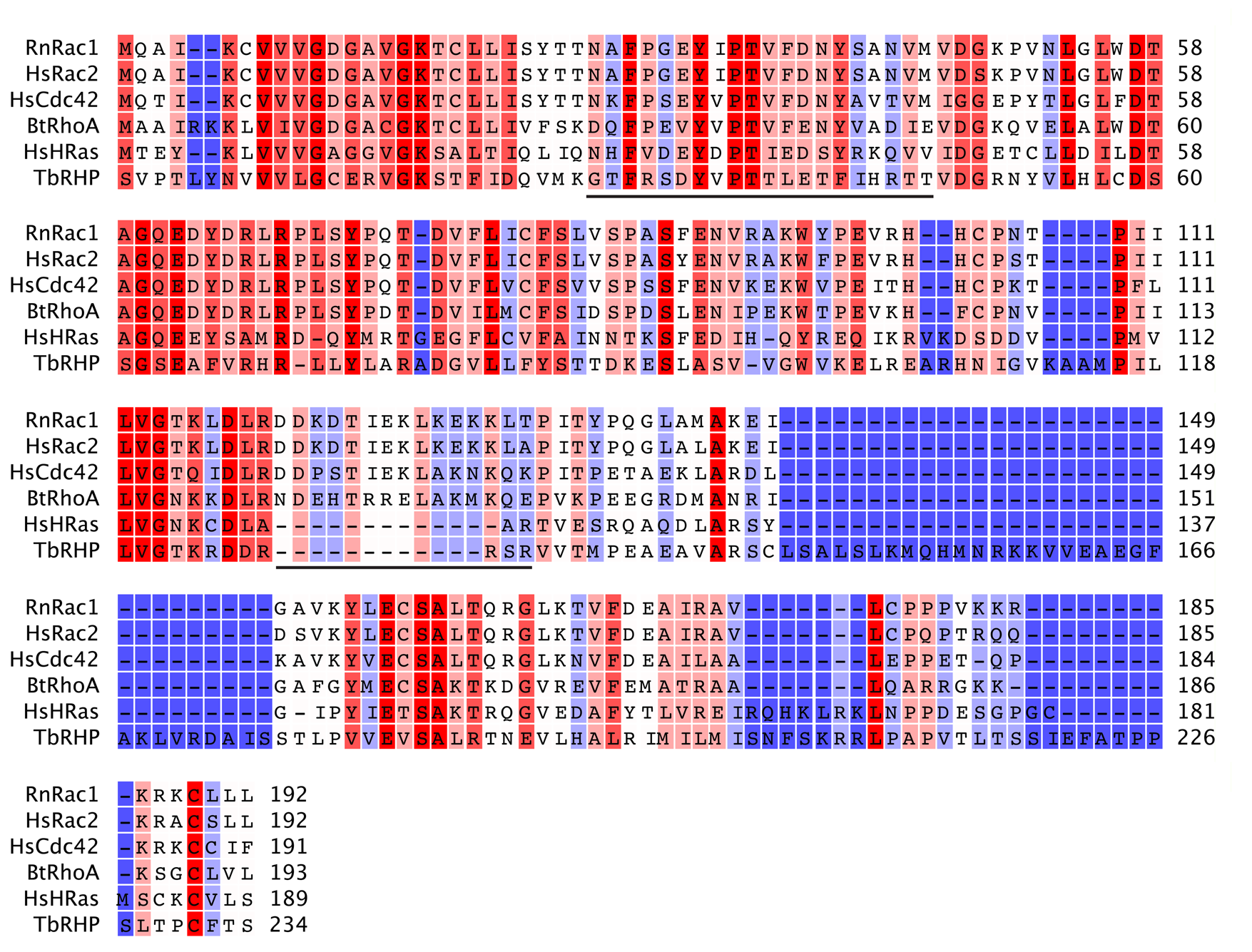

Supplement: Figure S1 — Alignment of TbRHP with representative Rho protein sequences. Predicted protein sequences were retrieved from the non-redundant database using BLASTp. Orthology was verified by reverse BLAST against the T. brucei genome sequence and selected sequences were aligned using ClustalX and default parameters. “-” represents gaps introduced into the alignment for optimization. Colorization indicates identity, conservative and semiconservative substitutions on a five point scale from red (conserved in 80% or more sequences), through light red, blue, light blue and white (no conservation). (TIF) [file pone.0026890.s001.tif]

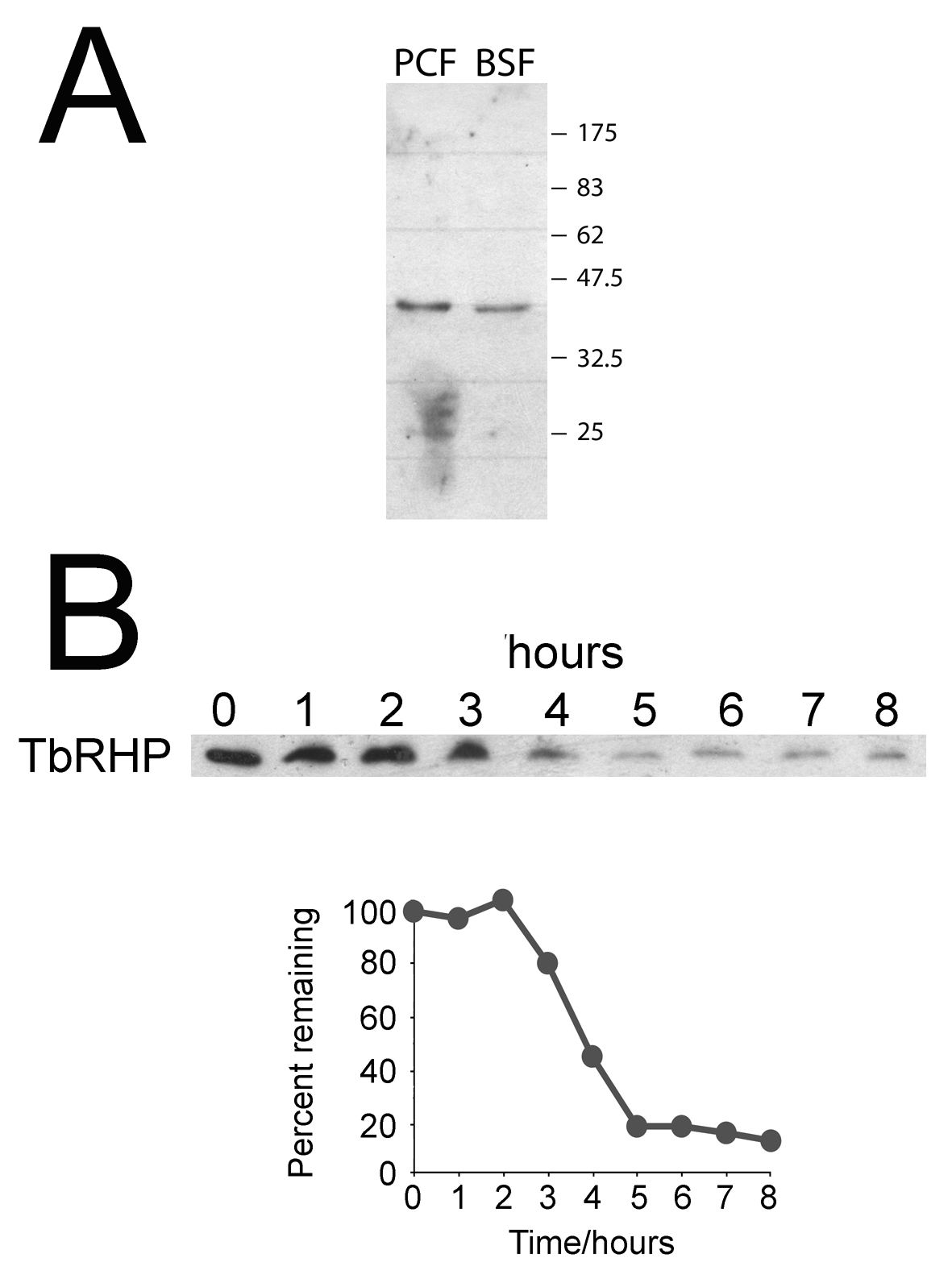

Supplement: Figure S2 — Characterization of anti-TbRHP antiserum and turnover of TbRHP. (A) Western analysis of whole cell lysates prepared from BSF and PCF trypanosomes (B and P respectively); Whole cell lysates from 107 cells were fractionated by SDS-PAGE and transferred to a nitrocellulose membrane. The blot was probed with affinity purified anti-TbRHP antibodies and signal was detected using ECL; a single band at ∼45 kDa was detected at equivalent intensity in both life stages. Numbers and bars at right indicate migration positions of co-electorphoresed molecular weight standards, in kDa. (B) Turnover of TbRHP in BSF cells. A log-phase BSF trypanosome culture was treated with cyclohexamide to inhibit new protein synthesis, and aliquots withdrawn from the culture at one hour intervals. Total lysates were fractionated by SDS-PAGE, transferred to nitrocellulose membrane and levels of TbRHP were monitored by Western blotting using affinity-purified anti-TbRHP antibodies. (C) Western blots were quantified using densitometry. TbRHP has a half-life of 3.5–4.0 hours. The data are a representative of three replicate experiments. (TIF) [file pone.0026890.s002.tif]

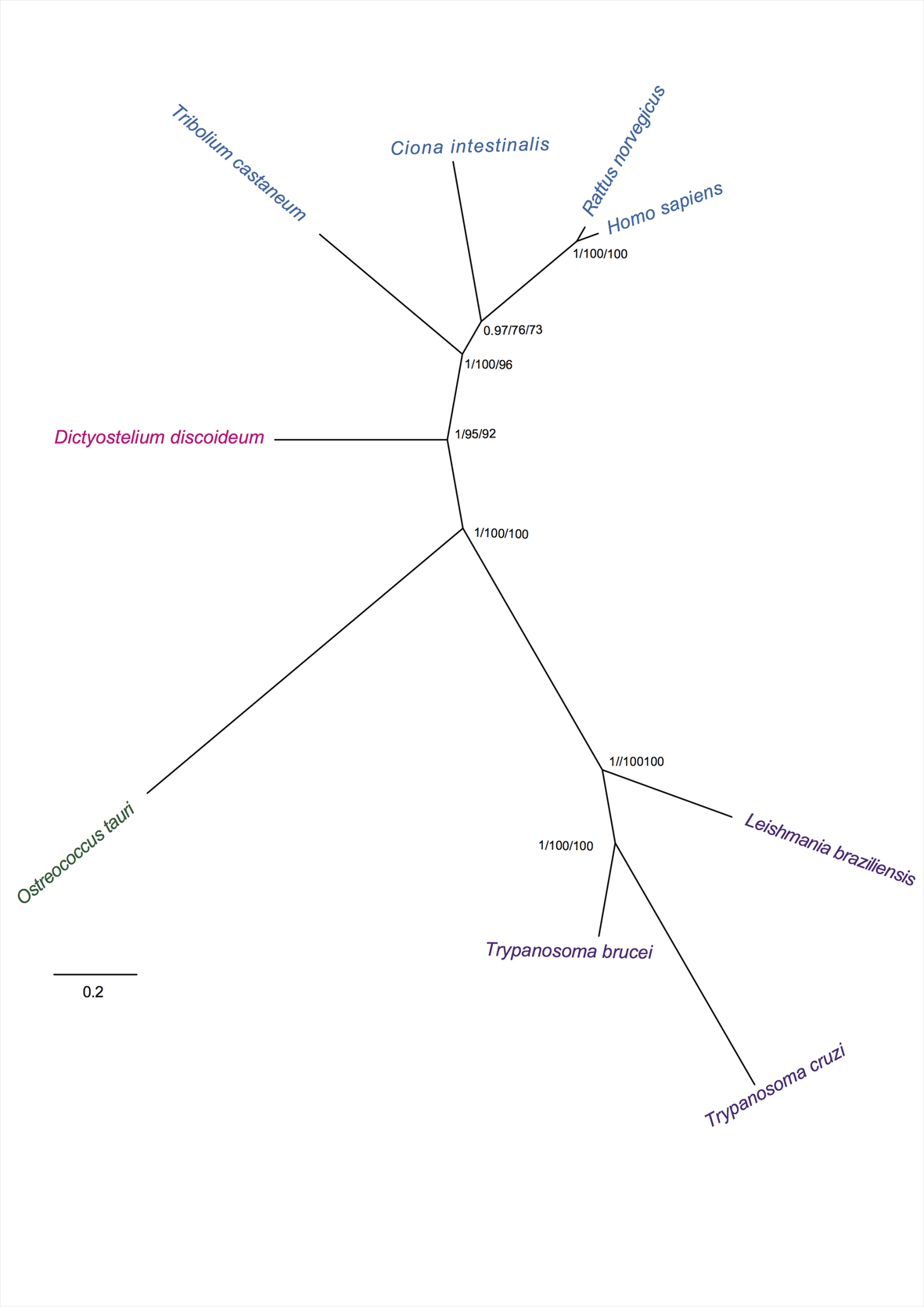

Supplement: Figure S4 — Phylogenetic reconstruction for OCRL family. Taxa included are as in (A). Taxon abbreviations are: Ci; Ciona intestinalis, Dd; Dictyostellium discoidium, Hs; Homo sapiens, Lb; Leishmania braziliensis, Ot; Ostreococcus tauri, Rn; Rattus norwegicus, Tb; Trypanosoma brucei, Tc; Trypanosoma cruzi and Tr; Tribolium castaneum. (TIFF) [file pone.0026890.s004.tif]

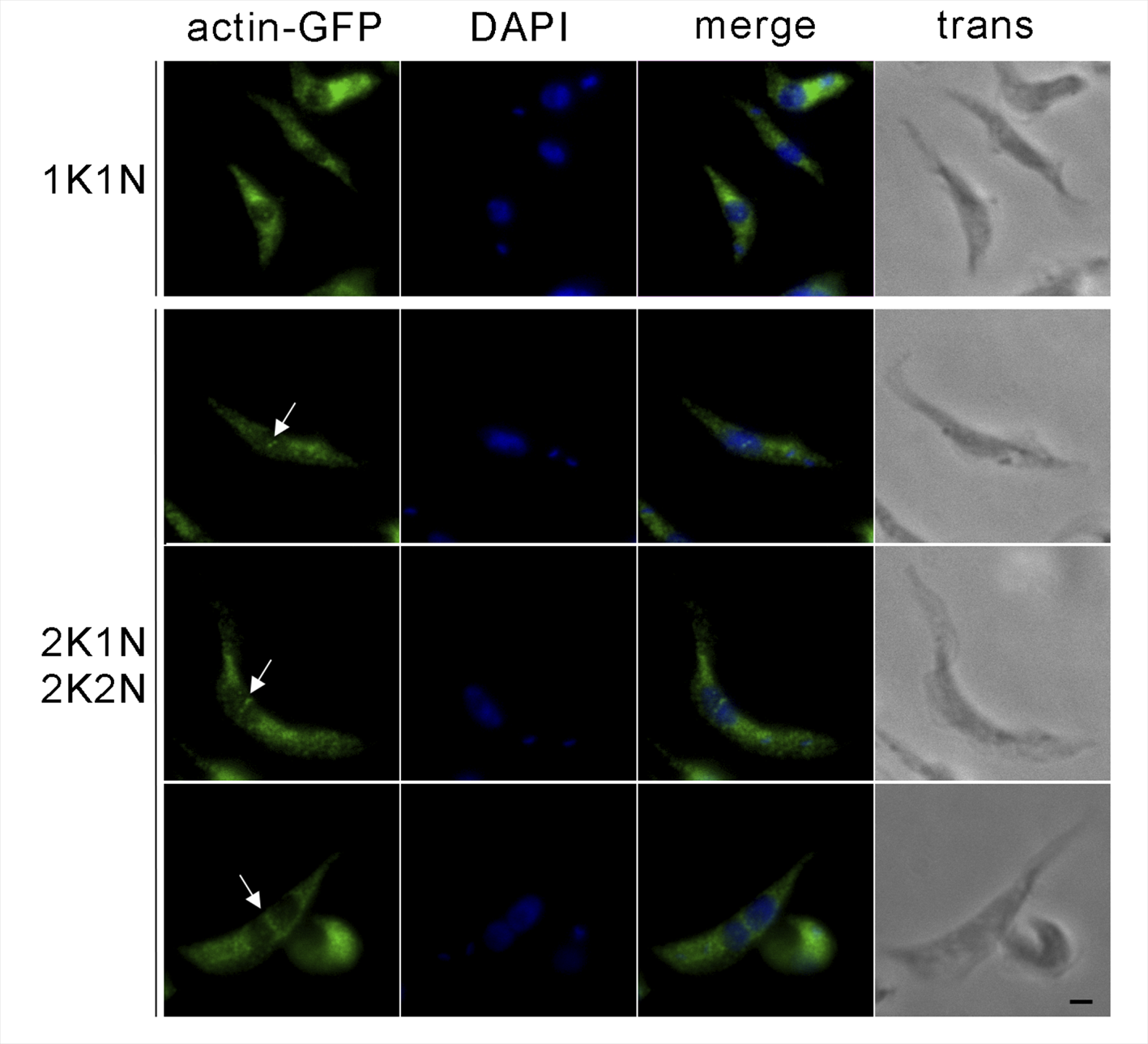

Supplement: Figure S5 — Actin localizes to the division plane between daughter nuclei in mitotic PCF cells. In cells expressing actin-GFP, a thin bridge of actin was observed at the division plane between dividing nuclei (white arrows) in mitotic cells (2K1N and 2K2N). This band of actin appeared to progressively thicken as nuclei divided. Such a structure was not observed in non-mitotic (1K1N) cells. (TIFF) [file pone.0026890.s005.tif]
