# Supplementary material for: A Novel Rho-Like Protein TbRHP Is Involved in Spindle Formation and Mitosis in Trypanosomes
Source: PLoS One. 2011 Nov 11;6(11):e26890. doi: 10.1371/journal.pone.0026890 (PMC3214021; doi:10.1371/journal.pone.0026890)
Supplement: Figure S3 — Tb09.160.4180 is a representative member of the OCRL family. Predicted protein sequences were retrieved from the non-redundant database using BLASTp. Orthology was verified by reverse BLAST against the T. brucei genome sequence and selected sequences were aligned using ClustalX and default parameters. “-” represents gaps introduced into the alignment for optimization, and “:”, “.” and “*” indicate semiconservative, conservative or identical amino acids respectively below the relevant system. Residues involved in inositol phosphate binding are indicated in bold and the position of the active arginine residue in the Rho GAP domain is shown bold underline, above the relevant system. Note that the O. tauri sequence is highly divergent within the Rho GAP domain, and is unlikely to be a member of the OCRL gene family. (RTF) [file pone.0026890.s003.rtf]

TriboliumcastaneumXP_972767         --------------------------------------------------CiXP_002122014                      --------------------------------------------------RnAAH98852|||Inpp5b                 --------------------------------------------------HsEAX07309|||inositolpolyphosp      MDQSVAIQETLAEGEYCPQAVQGVLCEGDSRQSRLLGLVRYRLEHGGQEHDdXP_647668                         --------------------------------------------------Tb09.160.4180||28G16.380|inosi      --------------------------------------------------Tc00.1047053505977.40|||inosit      --------------------------------------------------LbXP001563564                       --------------------------------------------------OtCAL51627                          --------------------------------------------------                                                                                      TriboliumcastaneumXP_972767         --------------------------------------------------CiXP_002122014                      ------------------------------MDKFTAELFQEHLSEFDPFARnAAH98852|||Inpp5b                 --------------------------------------------------HsEAX07309|||inositolpolyphosp      ALFLYTHRRMAITGDDVSLDQIVPVSRDFTLEEVSPDGELYILGSDVTVQDdXP_647668                         --------------------------------------------------Tb09.160.4180||28G16.380|inosi      --------------------------------------------------Tc00.1047053505977.40|||inosit      --------------------------------------------------LbXP001563564                       --------------------------------------------------OtCAL51627                          --------------------------------------------------                                                                                      TriboliumcastaneumXP_972767         --------------------------------------------------CiXP_002122014                      PTVQKVTRKPSRPPPPPPNVPPRRTNTVSEPLSQSALTTDLVNLDLDPKTRnAAH98852|||Inpp5b                 --------------------------------------------------HsEAX07309|||inositolpolyphosp      LDTAELSLVFQLPFGSQTRMFLHEVARACPGFDSATRDPEFLWLSRYRCADdXP_647668                         ------------------------------MGDIQNTDNIESNIDNNNNNTb09.160.4180||28G16.380|inosi      --------------------------------------------------Tc00.1047053505977.40|||inosit      --------------------------------------------------LbXP001563564                       --------------------------------------------------OtCAL51627                          --------------------------------------------------                                                                                      TriboliumcastaneumXP_972767         --------------------------------------------------CiXP_002122014                      EPKLVNVSKDDPPAS-----IMHLDNSGQKYPDLPRPKSSSFVSYISPT-RnAAH98852|||Inpp5b                 EADWEMSAGGGSR---------ERDCAGGYNVDSSRPNGRGLLPLDQSSGHsEAX07309|||inositolpolyphosp      ELELEMPTPRGCNSALVTWPGYATIGGGGSNFDGLRPNGKG-VPMDQSS-DdXP_647668                         NVSLESSSSSQNNNTN------TTTTTTTTSVDNLQVGVLSISDQSTPTITb09.160.4180||28G16.380|inosi      --------------------------------------------------Tc00.1047053505977.40|||inosit      --------------------------------------------------LbXP001563564                       --------------------------------------------------OtCAL51627                          -----------------------MVKTRSMRSTPEGTRAHPLAREWSSTM                                                                                      TriboliumcastaneumXP_972767         ----------MTDQ----SSDIP------VLRHQLAQGQAAS--------CiXP_002122014                      ---APKPMEIVTNQNNINSSDSSRAGMHDVGRSAFSNFEGSTGKNELTSHRnAAH98852|||Inpp5b                 ARCPDKPENSLTRQNRSKAEITDMVRSSTITVSDKAHILSMQKFG-----HsEAX07309|||inositolpolyphosp      -RGQDKPESLQPRQNKSKSEITDMVRSSTITVSDKAHILSMQKFG-----DdXP_647668                         ETPNQQQQQQQDDNNRGVSNETIKASLDGLKHNSLKTTFPNSTNHQYID-Tb09.160.4180||28G16.380|inosi      MVNGLPPAFQQTHYS-------SDDIFAFLRTASPVQSSCEILFP-----Tc00.1047053505977.40|||inosit      ---RFQGFLRRLMWN-------ATAWKMCLRRKAAPSGRMAICFRR----LbXP001563564                       MYPGLPRAFPNPEWQNVEDIFRQRTAFGFLAPNARASATADPAMA-----OtCAL51627                          HPSVVREAFLAESIDAYTTRVRTRVTTGTWNTNGKAPRGDEDVRGWLMG-                                                                                      TriboliumcastaneumXP_972767         --NRESILRYQLKLKEPEYTQQQEFSIFVGTWNVNGQPPSVSLRPWLSFDCiXP_002122014                      LLERDKLMPLYMAKRQDDYTSTAPFNVFVGTWNVNGQLPTEPLDAWVSSERnAAH98852|||Inpp5b                 --LRDTMVRSHLLQKEGEYTYIQNFRFFVGTYNVNGQSPKECLRPWLSSDHsEAX07309|||inositolpolyphosp      --LRDTIVKSHLLQKEEDYTYIQNFRFFAGTYNVNGQSPKECLRLWLSNGDdXP_647668                         --VNTQWITNKLKERESEFTEKRGMSIFLGTWNVNGKKPSESLDPWLKDPTb09.160.4180||28G16.380|inosi      ---EEVWVQQELQFYEDSYTEQQDLRTCIVTFNVASKKPPQNLASLIALTTc00.1047053505977.40|||inosit      ---KNRGCSGSCSTMRICTRNSRNLRCVLSRSTLRARSHLQIWPPSFRLKLbXP001563564                       ---ADAWVQREIGYYEVNYTTVEDLSVCVTSFNVGCKKPVLPLTSLVCLTOtCAL51627                          -ARGEEGTPDVVVVGFQEIVPLHVDKVLAGKDAAATEAWEEVLDRALNGD                                                                        .         .   TriboliumcastaneumXP_972767         EE----------PPDLYAIGLQEIDLSKEAFLFNDTPREAEWVKYVMDGVCiXP_002122014                      AT----------PPDVYAIGFQELDLSKEAFVFTESSREEEWMRAVKQALRnAAH98852|||Inpp5b                 TK----------APDVYCVGFQELDLSKEAFFFHDTPKEEEWFKAVSESLHsEAX07309|||inositolpolyphosp      IQ----------APDVYCVGFQELDLSKEAFFFHDTPKEEEWFKAVSEGLDdXP_647668                         SMS--------LQPDIYAIGFQELDLTAEALLLGDTTRSLPWEQHILNTLTb09.160.4180||28G16.380|inosi      MPGAGGE-----PVDLIMVSLQEVDMSASAMLKDETDASVVWVSALQAVITc00.1047053505977.40|||inosit      WRRTQTG-----PWTS-WSAFRKLTAPLPCRRKRKHLHHGSWDSTPLWAQLbXP001563564                       SSGGGTDDAPARPTDLIVVGMQEVDMSATALFKQETEAASPWVAGLNAAIOtCAL51627                          AARQGATK----RRGTMVDYDASYGNDAGRGGGASTSGTTSWVSFDDADV                                                          .           .      *        TriboliumcastaneumXP_972767         HSK---------------AKYKSVAVTRLVGMQLIVLVNSKHYQFVKNVACiXP_002122014                      HPG---------------ASYKLVKHIRLVGMMLLVFAQDRHLQDITAVARnAAH98852|||Inpp5b                 HPD---------------AKYAKVKLIRLVGIMLLLYVKQEHAAYISEVEHsEAX07309|||inositolpolyphosp      HPD---------------AKYAKVKLIRLVGIMLLLYVKQEHAAYISEVEDdXP_647668                         QG-----------------DYVKLLSKQLVGILLCVYVKKEHKPHIANVQTb09.160.4180||28G16.380|inosi      GAD---------SQAAGESPYFAFPPKQLVGLLLCVFIRRSLLPHAQKMATc00.1047053505977.40|||inosit      TLG---------HQAT--PHILPFHPSNLLASYYVSTYDANFFPTCRRCLLbXP001563564                       GADSSNSTATSSSGGAGASPYYAFPPKQLVGLLLCVFIRRPLLSAVSEFSOtCAL51627                          SRR---------AKSKAEPTYRPVAQKQLVGVYITVWVKTSLLPHLKDVR                                                           .   .*:.                   TriboliumcastaneumXP_972767         VDTVGTGL---LGKMGNKGGVAVRLELHNTS-LCFVNCHLAAHIE--EFECiXP_002122014                      SHHVGTGL---LGKMGNKGGVGIRIMLHNSS-LCFINSHLAAHLE--EIQRnAAH98852|||Inpp5b                 AETVGTGI---MGRMGNKGGVAIRFQLHNTS-ICVVNSHLAAHTE--EYEHsEAX07309|||inositolpolyphosp      AETVGTGI---MGRMGNKGGVAIRFQFHNTS-ICVVNSHLAAHIE--EYEDdXP_647668                         SDIAAVGI---MGMMGNKGGVAIRFSFYNTT-ICILNSHLNAHMD--NVLTb09.160.4180||28G16.380|inosi      ITTVATGA---LGTMGNKGAVGLHLGLCRSN-LCFINMHLAAGQK--NVVTc00.1047053505977.40|||inosit      LRLLRQER---WVAWEIK--VLLVFVLCYTAPACALMFTLLVR----VMWLbXP001563564                       MATVATGA---LGSMGNKGAVGFHLVLHRTS-ICVITAHLAAGHD--NVROtCAL51627                          VASVATGFNIGVGVLGNKGACAVWMKLYSTP-LVFVCSHLSAGTKPGDEH                                                     *    . : :  :     :   * .        TriboliumcastaneumXP_972767         RRNQDYKDINARINFRK-----------------------QPQSIKDHEQCiXP_002122014                      RRNQDYEDICSRMKFPDPEG--------------------QSISVFSNDVRnAAH98852|||Inpp5b                 RRNQDYRDICSRMQFSQVDPS------------------LPPLTISKHDVHsEAX07309|||inositolpolyphosp      RRNQDYKDICSRMQFCQPDPS------------------LPPLTISNHDVDdXP_647668                         RRNQDMKDISKNIKFINESSTD-----------------HSTINIFDHDQTb09.160.4180||28G16.380|inosi      KRNNDVSKIFMGMDFNTTKRPISLETRGGNSAQSELQFQYPEFLPHNNDVTc00.1047053505977.40|||inosit      KRNADASSIFTGMDFNVQKRQALVASVEDGYIISEYMDQNPELRPHDHDILbXP001563564                       KRNEDINTIFRSMDFNAARRAETQMSASPNAPIDESAFL--ELYPRDHDIOtCAL51627                          RRNEDFATIVDQLRFHPPDGLDG-----------------VEHTIEDAAS                                    :** *   *   : *                               .                                                RTriboliumcastaneumXP_972767         VYWLGDLNYRITDLNTQQVKTLLARNEIVTLLKADQLNQQKD-RGHVLLDCiXP_002122014                      LIWLGDLNYRIDKLPLETVKERIEKGQYEKLKEHDQLTNQRR-EGNVYPDRnAAH98852|||Inpp5b                 ILWLGDLNYRIEELDVEKVKKLVEEKAFHTLYAHDQLKIQVA-AKTVFEGHsEAX07309|||inositolpolyphosp      ILWLGDLNYRIEELDVEKVKKLIEEKDFQMLYAYDQLKIQVA-AKTVFEGDdXP_647668                         LFWIGDLNYRIP-LPDNEVKEKIKKKDFYNLFLVDQLNQQMK-AGAVFEGTb09.160.4180||28G16.380|inosi      IVVAGDLNYRVN-LTYRESLQLAMKKDYATLLKHDEFVKELANTHSPWMGTc00.1047053505977.40|||inosit      IIVSGDLNYRTK-LNYEEALELATRCDTERLLKHDELVSELANSHSPWWGLbXP001563564                       IIVAGDLNYRLR-LPYETAVHLANSGQFSELLAHDQLAAEMKNPHTPWLNOtCAL51627                          AIWMGDLNYRLN-ATDKFVRDCITKGTFSQLLACDQLNIERA-GGRVFQG                                        ******      .             *   *::  :         .                                                KY                             RTriboliumcastaneumXP_972767         YTEGDITFHPTYKYDLNTDTFD----------------TSEKARPPAWTDCiXP_002122014                      FEEGDLTFRPTYKYNPGTDEWD----------------TSEKCRCPAWCDRnAAH98852|||Inpp5b                 FTEGEITFQPTYKYDTGSDNWD----------------TSEKCRAPAWCDHsEAX07309|||inositolpolyphosp      FTEGELTFQPTYKYDTGSDDWD----------------TSEKCRAPAWCDDdXP_647668                         FQEPPISFAPTYKYDAGTEEYD----------------SSEKKRTPAWCDTb09.160.4180||28G16.380|inosi      FVELTPTYPPTYRYDIGTNNYD----------------TSEKQRVPSYTDTc00.1047053505977.40|||inosit      FVDLTPTFPPTYRYDIGTNIYD----------------TSEKQRVPSYTDLbXP001563564                       FINFTPTHMPTYRFDIGTDVYD----------------TSEKRRIPSYTDOtCAL51627                          WHESELTFAPTYKYRPGTNIYSGAEDADADVVDAGQKKEEEKKRTPAWCD                                    : :   :. ***::  .:: :.                 .** * *:: *                                                                        HTriboliumcastaneumXP_972767         RILWRGEG------------IYQTAYRSHMDVRISDHKPVSALFKSEISVCiXP_002122014                      RILWKESAKAKPRKTKRKDLVQLLKYQAHLSLKISDHKPVSAVFKINVKVRnAAH98852|||Inpp5b                 RVLWRGKN------------ISQLSYQSHMSLKTSDHKPVSSVFEIGVRVHsEAX07309|||inositolpolyphosp      RILWKGKN------------ITQLSYQSHMALKTSDHKPVSSVFDIGVRVDdXP_647668                         RILWKTHKKAEN--------VGILSYK-RAELISSDHRPVSASFVIKIKVTb09.160.4180||28G16.380|inosi      RIAIWTRRRDHQS------SIRLERLQALTDVMSSDHKPVQACLCLPISRTc00.1047053505977.40|||inosit      RIVTWTKRKSHQK------LIIVERLQAIVDIFSSDHKPVQALLRLPILCLbXP001563564                       RICVWSRRKSMES------RIRLDRISALMEVRSSDHKPVQALARIPVSVOtCAL51627                          RVLWNGDFD-----------INLLEYG-RSELTHSDHKPVHAVFSIVVRE                                    *:                  :          :  ***:** :     :  TriboliumcastaneumXP_972767         IDQNKFRRVHEDLLKKMDK--LENEFLPQVMVDQTEVVFDLVKFREP-QACiXP_002122014                      INRELYRKVYEEEIRRLDR--MENEWLPTMVLNQHSLEFGTVKFQQA-VQRnAAH98852|||Inpp5b                 VNEELYRKTLEEIVRSLDK--MENANIPSVTLSKREFCFENVKYMQL-QTHsEAX07309|||inositolpolyphosp      VNDELYRKTLEEIVRSLDK--MENANIPSVSLSKREFCFQNVKYMQL-KVDdXP_647668                         VIPDSKNRIYQEIVKELDK--KENDSMPDANISTNMVDFETIKFMQP-ISTb09.160.4180||28G16.380|inosi      EVLEKKISVTQSLRDSVKREGLDRIRKAKISVNSQSLNFGVRQFGDCGSRTc00.1047053505977.40|||inosit      EVEEKKKNVTQLLNDRIAQVGLDRSTSAKTTISPSLLDFGEQRFYECGARLbXP001563564                       EVPAQKAQIVSSLREKVATIGLAQASSAKISLSMSKVNFQAQCFHNCGTQOtCAL51627                          LDPQKLNALMFDLRRRLDH--VEMAAQPKCAIVNPSVDLG-EMFYSRDAL                                                    :          .   :    . :    : .    TriboliumcastaneumXP_972767         REIIIANTGQVPAEFEFIKKLDEATYCKDWLRITPFCGTIDPGDKCDIKFCiXP_002122014                      RTVEILNTGQTPCHFEFIGKLGEKAFCKPWLTVGKPKGYVLPGDKLVIEFRnAAH98852|||Inpp5b                 ATFTIHN-GQVPCEFEFINKPDEETYCKQWLTAKPSRGFLLPDSHVEIELHsEAX07309|||inositolpolyphosp      ESFTIHN-GQVPCHFEFINKPDEESYCKQWLNANPSRGFLLPDSDVEIDLDdXP_647668                         KQLIFENIGQVIARFQFIPKLDETILCKPWLKISPLAGMMIPKEKVTIDLTb09.160.4180||28G16.380|inosi      QPLKITNEGDCVAVIKAFRQQDGDPSKGAWLRVFPLIIFIPPRKEKEVMITc00.1047053505977.40|||inosit      RVLSLTNVGECVALVKVFRQRNNDISEGAWLRVYPSNFSILPGEKKEVTVLbXP001563564                       EVVTVQNNGNCVAVVRVVRQREGDYSEGSWLRVTPQELAILPGESQDVQIOtCAL51627                          GEFTMSNVGDVPAKFSLVSPIPGGPATPGWITVNPMAGSLLPGEEIVLKV                                      . . * *:  . .  .           *:        : * .   : .TriboliumcastaneumXP_972767         EVNLER---------------------------EMDKVYDILVLHLKGGKCiXP_002122014                      EVYVNKNTA-------------------PSLNKEEDKIEDILILHLVGGKRnAAH98852|||Inpp5b                 ELFVNKATA-------------------TKLNSGKDTIEDILVLHLHRGKHsEAX07309|||inositolpolyphosp      ELFVNKTTA-------------------TKLNSGEDKIEDILVLHLDRGKDdXP_647668                         TIYVDNLTSGLFNIN------------NNSTNSTNESMDDILILHLENGKTb09.160.4180||28G16.380|inosi      ECQLDRNSTEWVRNW------------RPFEGRGEVEITSTLVLCVRNGDTc00.1047053505977.40|||inosit      ECQLHPRCMRWMGSW------------RPFEGRGRLSLSSMLLVFVNQGSLbXP001563564                       ETAFHPRCTLWMAAW------------RPYQGRGSIELSSVLLFCCRNGPOtCAL51627                          RACVQGGRESGPSAFSHDSLSRRSEEPLIEIGAKPKLVEAVLVVRLEGGR                                       ..                                :   *:.    * TriboliumcastaneumXP_972767         DMFIIVTGECQRSCFTSSISTLCRAPVPLLQMSEEQRKQAENMESR----CiXP_002122014                      DFFITVNGKYSPSCFGTSIEALCWMKKPIEQMDRKELTRLSCTESRDWQVRnAAH98852|||Inpp5b                 DYFLSVTGNYLPSCFGSPIHTLCYMREPILDLPLKIVNELTLMTVQTA--HsEAX07309|||inositolpolyphosp      DYFLSVSGNYLPSCFGSPIHTLCYMREPILDLPLETISELTLMPVWTG--DdXP_647668                         DYFISISGKFQKTCFGNTLDNLVRYPHPIRNN-------LPIPPEQ----Tb09.160.4180||28G16.380|inosi      IHFVECRCTVRPSVFGNTLDNISLLRNEVCAAAYTLWGTPERRSGG----Tc00.1047053505977.40|||inosit      VHLVECICTFNPSVFGNSLENISLLGNEACLTAYGIKGDLEKLRKV----LbXP001563564                       VQAVECQCVLSPSVFGNALENIALLQDTPCVEAYAQKADFEEVVRQ----OtCAL51627                          DFFVMVKGQYIPCVFGTPLEDLPTTMFP----------------------                                       :          * ..:  :                                                                                                   RTriboliumcastaneumXP_972767         --------VLYSIPRELWHLVDNLYRYGLKTRDLFESCAL----HEEIIRCiXP_002122014                      NNEKHHSSQMLNVPKELWRLLDHLYQYARHQPDLFQQPGL----HEELKMRnAAH98852|||Inpp5b                 -DDRSQLEKPMEIPKELWMMVDYLYRNAVQQEDLFQQPGL----RPEFEHHsEAX07309|||inositolpolyphosp      -DDGSQLDSPMEIPKELWMMVDYLYRNAVQQEDLFQQPGL----RSEFEHDdXP_647668                         --------KKLSIPKELWRIIDYIYYNGLKEEGLFIKSGV----TKEMELTb09.160.4180||28G16.380|inosi      --------CMPQIPKELWYLCEAIYERGAQQPNLFTENPS----TEVCDATc00.1047053505977.40|||inosit      --------VRPQLPKELWFLCEAIYARGARQPNLFTENGS----AEACTELbXP001563564                       --------VRPHVPKELWYLVYVIAQH-PREPGLFTRSTN----KEVCRHOtCAL51627                          ----------PNCPQVISTLVDYVFENISTAPGIFFEPLDGLRTPGGVAK                                                 *: :  :   :        .:*               TriboliumcastaneumXP_972767         IRDWLD---YGSVDS-LPGTVQAVAEALLLFLSYTKDPIVPFELHDSCIACiXP_002122014                      IQENLDS-QLPTVGTPLPGSNHSVAEALLIFLEALPEPVIPFEYYNMCLQRnAAH98852|||Inpp5b                 IRDCLD---TGMIDH-LCANNHSVAEALLLFLESLPEPVICYSAYHSCLEHsEAX07309|||inositolpolyphosp      IRDCLD---TGMIDN-LSASNHSVAEALLLFLESLPEPVICYSTYHNCLEDdXP_647668                         IRDCLD---TAEPFSSISFSIHSMAETLIRFLESLVEPVIPFNMYQQALDTb09.160.4180||28G16.380|inosi      IMKHLN---TQCRPLPSEYNVQCISACLIYFLQSLQEPVVPYELYEKALATc00.1047053505977.40|||inosit      VMRHLD---TCCEPIPAEFDIQSVATCFITFLQSLQEPVVPFSLYGAAIALbXP001563564                       IMELLD---TKNAALPVDTDVHCAAECLLAFLKNLREPVVPYAQYAAALAOtCAL51627                          IAETLQHGETDIDFGALGVNVYDVGEALVSLLSALPRRILDEPGVLDAVD                                    :   *:                  .  :: :*.     ::       .: TriboliumcastaneumXP_972767         AANNFQNC-----RLIIQQKMSDVHRNVFLYICMFLKELLKYSN--ENGYCiXP_002122014                      LYNNYDQG-----QELIH-KMPPHHVNVFNYIISFLQELPKYE---ENGLRnAAH98852|||Inpp5b                 CSGSHTAS-----KQVIS-TLPTFHKNVFSYLMAFLQELLKNSA--KNHLHsEAX07309|||inositolpolyphosp      CSGNYTAS-----KQVIS-TLPIFHKNVFHYLMAFLRELLKNSA--KNHLDdXP_647668                         ASSSPLSC-----KTLVS-HLPSVNYNVFFYLISFLIETLSNQK--ENDLTb09.160.4180||28G16.380|inosi      VLKSKSGNP----FQFVQQQLPPLHANVWIYVCSLMNFLLRPVNTCGNGLTc00.1047053505977.40|||inosit      AGKAGGRAP----LVFVQQQLPPQHANVWIYVCALLNFLLRPVNARSNELLbXP001563564                       AGRAKGKAP----LQFLR-QLPTMHANVWLYVLSLLNYLLRPVNSSDNELOtCAL51627                          AMKLDDPQPKEFAHGLLGRHLSTKAKAALVHIAALIKRLDEECAKTGARV                                                   .:   :.     .  ::  ::              TriboliumcastaneumXP_972767         DAKTLASLFGDILLR-DPIRNS-------------------------KPQCiXP_002122014                      NLHLLATIFSGLLLRPSSVQRK-------------------------QNTRnAAH98852|||Inpp5b                 DENILASIFGSLLLRSPAGHRK-------------------------LDMHsEAX07309|||inositolpolyphosp      DENILASIFGSLLLRNPAGHQK-------------------------LDMDdXP_647668                         KPDQLAIIFSTVLLRPSPQSQL-------------------------SQFTb09.160.4180||28G16.380|inosi      TTKFLARVLSDVMLVRPEALTQMPPSVGTCTHQEVASNAPPKGGTALQAFTc00.1047053505977.40|||inosit      TPRILAKLFSEVMLVRPGVLIHDSSGHREGSYNGIA--APPQGVLTSQPLLbXP001563564                       DAPLLAHIFSAVLIGRPTDAQGAIPARLQ------------QGGGVDQQVOtCAL51627                          HVTEMILTFATCLFPESTEQPNP----------------------RRVAF                                        :   :.  ::                                    TriboliumcastaneumXP_972767         --ANRGKANFVYNFLVNDLSSSIIPNK--------CiXP_002122014                      --EGRMDRMKATSFIEMFLKKKLIE----------RnAAH98852|||Inpp5b                 --AEK---KKAQEFIHHFLCNPL------------HsEAX07309|||inositolpolyphosp      --TEK---KKAQEFIHQFLCNPL------------DdXP_647668                         PPDATTVKKKADLILHFLISKDLIN----------Tb09.160.4180||28G16.380|inosi      RQQLQQEREDALRFVECFLVPPPAVIL--------Tc00.1047053505977.40|||inosit      RLQLQQEKEDAMSLIEYFLMPPPESLR--------LbXP001563564                       RQQLQQESDDALALVEYFLSTPPSMLAGVE-----OtCAL51627                          IGALAGCHPSFLRTLRAREVAPPPSEPSFLPGVDR                                                  :                    
