# Supplementary material for: A Novel Rho-Like Protein TbRHP Is Involved in Spindle Formation and Mitosis in Trypanosomes
Source: PLoS One. 2011 Nov 11;6(11):e26890. doi: 10.1371/journal.pone.0026890 (PMC3214021; doi:10.1371/journal.pone.0026890)
Supplement: Table S1 — Accession numbers for sequences used in this study. Top: Rho and Rho-related proteins included in reconstruction in Figure 1. Middle: OCRL proteins included in the analysis of OCRL phylogeny in Figure S2. Lower: TbRHP syntenic genes and orthologues from trypanosomes and Leishmania species. (PDF) [file pone.0026890.s006.pdf]

| Tree figure name | Accession No. | Abbreviation |
| --- | --- | --- |
| Homo sapiens RhoA | NP_001655 | HsRhoA |
| Homo sapiens RhoB | NP_004031 | HsRhoB |
| Homo sapiens RhoC | NP_786886 | HsRhoC |
| Homo sapiens RhoD | NP_055393 | HsRhoD |
| Homo sapiens RhoE | NP_005159 | HsRhoE |
| Homo sapiens RhoF | NP_061907 | HsRHOF |
| Homo sapiens RhoG | AAM21121 | HsRhoG |
| Homo sapiens RhoH | NP_004301 | HsRhoH |
| Trypanosoma cruzi Rho | XP_805843 | TcRho |
| Saccharomyces cerevisiae Ras | AAA34959 | ScRas |
| Cryptococcus neoformans Ras | XP_566685 | Cnm08040 |
| Homo sapiens Ras | CAI18827 | HsRas |
| Naegleria gruberi Ras1 | fgeneshHS_pm.scaffold_48000003 | NgGTP2 |
| Phytophthora sojae Ras | fgenesh1_pm.C_scaffold_70000008 | Pr72012 |
| Saccharomyces cerevisiae RLP | EDN61740 | ScRLP |
| Homo sapiens Rap | NP_056461 | HsRLP |
| Entamoeba histolytica Rap | XP_651163 | Eh00077 |
| Dictyostelium discoideum Ras | XP_646561 | DDB0229439 |
| Trichomonas vaginalis Ras1 | XP_001327171 | Tv93986 |
| Trichomonas vaginalis Ras2 | XP_001580142 | Tv86622 |
| Naegleria gruberi Ras2 | fgeneshNG_pg.scaffold_26000199 | NgGTP3 |
| Trypanosoma brucei RLP | XP_828678 | TbRLP |
| Trypanosoma brucei RHP | XP_822866 | TbRHP1 |
| Trichomonas vaginalis Cdc42 | XP_001315127 | Tv85938 |
| Naegleria gruberi Cdc42 | fgeneshHS_pm.scaffold_27000004 | NgCdc42 |
| Homo sapiens Cdc42 | CAI19851 | HsCdc42 |
| Saccharomyces cerevisiae Cdc42 | NP_013330 | ScCdc42 |
| Phytophthora sojae Cdc42 | fgenesh1_pm.C_scaffold_1384000001 | Pr72470 |
| Dictyostelium discoideum Rho | XP_640169 | DD0204884 |
| Entamoeba histolytica Rho | XP_656745 | EhRho |
| Arabidopsis thaliana Rop4 | AAC78242 | AtRop4 |
| Saccharomyces cerevisiae Rho4 | NP_012981 | ScRho |
| Naegleria gruberi RLJ | gw1.3.204.1 | NgGTP1 |
| Phytophthora sojae RLJ | fgenesh1_pm.C_scaffold_20000001 | Pr71443 |
| Homo sapiens RLJ | NP_057628 | HsRLJ |
| Chlamydomonas reinhardtii RLJ | estExt_gwp_1W.C_240174 | Cr137686 |
| Trypanosoma brucei RLJ | XP_829353 | TbRLJ |
| Trichomonas vaginalis RLJ | XP_001330169 | Tv93909 |
| **ORCL/RhoGAP accessions** | | |
| Trypanosoma brucei | Tb09.160.4180 | TbORCL |
| Trypanosoma cruzi | Tc00.1047053505977.40 | TcORCL |
| Leishmania braziliensis | XP_001563564 | LBORCL |
| Dictyostelium discoideum | XP_647668 | DdORCL |
| Rattus norvegicus | AAH98852 | RnInpp5b |
| Homo sapiens | EAX07309 | HsORCL |
| Ciona intestinalis | XP_002122014 | CiORCL |
| Ostreococcus tauri | CAL51627 | OtORCL |
| Tribolium castaneum | XP_972767 | TrcaORCL |
| **Trypanosoma RHP accessions** | | |
| Trypanosoma brucei | RHP | Tb927.10.6240 |
| Trypanosoma brucei gambiensie | RHP | Tbg972.10.7620 |
| Trypanosoma congolense | RHP | TcIL3000.10.5300 |
| Trypanosoma congolense | RHP (truncated) | TcIL3000.10.5310 |
| Trypanosoma vivax | RHP | TvY486_1006200 |
| Trypanosoma cruzi | RHP | Tc00.1047053507641.104 |
| Leishmania braziliensis | RHP | LbrM.35.2010 |
| Leishmania infantum | RHP | LinJ.36.1900 |
| Leishmania major | RHP | LmjF.36.1820 |
| Lesihmania mexicana | RHP | LmxM.36.1820 |
